# ^111^In-labelled polymeric nanoparticles incorporating a ruthenium-based radiosensitizer for EGFR-targeted combination therapy in oesophageal cancer cells†
†Electronic supplementary information (ESI) available: Supplementary figures and tables. See DOI: 10.1039/c7nr09606b


**DOI:** 10.1039/c7nr09606b

**Published:** 2018-05-29

**Authors:** Martin R. Gill, Jyothi U. Menon, Paul J. Jarman, Joshua Owen, Irini Skaripa-Koukelli, Sarah Able, Jim A. Thomas, Robert Carlisle, Katherine A. Vallis

**Affiliations:** a CRUK/MRC Oxford Institute for Radiation Oncology , Department of Oncology , University of Oxford , Oxford , UK . Email: Katherine.vallis@oncology.ox.ac.uk; b Institute of Biomedical Engineering , Department of Engineering Science , University of Oxford , Old Road Campus Research Building , Oxford OX3 7DQ , UK; c Department of Chemistry , University of Sheffield , Sheffield , UK

## Abstract

Radiolabelled, drug-loaded nanoparticles may combine the theranostic properties of radionuclides, the controlled release of chemotherapy and cancer cell targeting. Here, we report the preparation of poly(lactic-*co*-glycolic acid) (PLGA) nanoparticles surface conjugated to DTPA-hEGF (DTPA = diethylenetriaminepentaacetic acid, hEGF = human epidermal growth factor) and encapsulating the ruthenium-based DNA replication inhibitor and radiosensitizer Ru(phen)_2_(tpphz)^2+^ (phen = 1,10-phenanthroline, tpphz = tetrapyridophenazine) **Ru1**. The functionalized PLGA surface incorporates the metal ion chelator DTPA for radiolabelling and the targeting ligand for EGF receptor (EGFR). Nanoparticles radiolabelled with ^111^In are taken up preferentially by EGFR-overexpressing oesophageal cancer cells, where they exhibit radiotoxicity through the generation of cellular DNA damage. Moreover, nanoparticle co-delivery of **Ru1** alongside ^111^In results in decreased cell survival compared to single-agent formulations; an effect that occurs through DNA damage enhancement and an additive relationship between ^111^In and **Ru1**. Substantially decreased uptake and radiotoxicity of nanoparticles towards normal human fibroblasts and oesophageal cancer cells with normal EGFR levels is observed. This work demonstrates nanoparticle co-delivery of a therapeutic radionuclide plus a ruthenium-based radiosensitizer can achieve combinational and targeted therapeutic effects in cancer cells that overexpress EGFR.

## Abstract 2

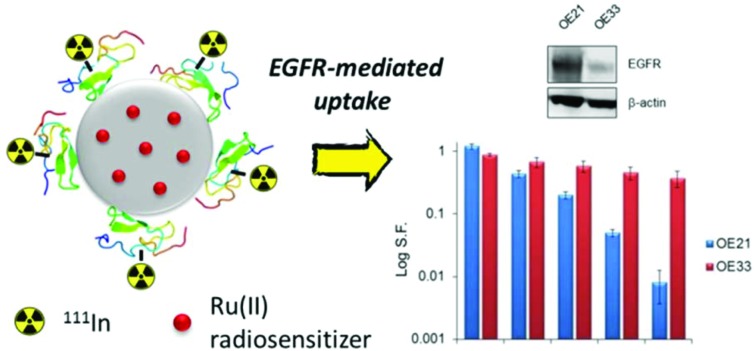
EGFR-targeted PLGA nanoparticles co-deliver the Auger electron emitter ^111^In and a ruthenium(ii) radiosensitizer for combined therapeutic effects.

## Introduction

Oesophageal cancer is an aggressive and highly lethal form of cancer.^1^ Current non-surgical treatments are based on the use of radiotherapy in combination with cisplatin DNA-damaging chemotherapy, usually alongside the antimetabolite fluorouracil (5FU). However, survival rates remain poor and attempts to improve patient outcomes with alternative chemotherapy have been unsuccessful.^1^ Advances in the identification of key genetic and epigenetic alterations underlying the development and progression of cancer have paved the way for targeted therapy as an alternative to cytotoxic chemotherapy. A commonly proposed molecular target is epidermal growth factor receptor (EGFR), a plasma membrane-bound receptor overexpressed by >67% of oesophageal primary tumours.^2^ In addition, EGFR overexpression correlates with poor patient survival following chemoradiotherapy^3^ and radioresistance.^4^ Unfortunately, clinical trials of EGFR inhibitors have demonstrated limited benefit to date.^5^ Hence, there remains an urgent need to develop new therapeutics active towards EGFR-overexpressing oesophageal cancers.

Nanoparticles surface labelled with radionuclides can combine the therapeutic properties of ionizing radiation (IR) with the controlled release of radiosensitizing chemotherapy.^6^–^9^ By employing targeting moieties designed to bind a cell-surface receptor overexpressed by cancerous but not normal cells, this may enhance delivery of the therapeutic combination to cancer cells, boosting therapeutic efficiency.^10^ A potential advantage of this strategy over external beam radiotherapy is that distant metastases in addition to primary tumour(s) may be targeted. Although the majority of clinical therapeutic radionuclide therapeutics are low LET (linear energy transfer) β-emitters (LET = 0.1–1.0 keV μm^–1^),^11^ high LET Auger electrons (LET = 4–26 keV μm^–1^) emitted by radionuclides such as ^111^In are also of interest in this context.^12^ The short range of Auger electrons (<20 nm in biological media) means cellular internalisation is paramount for radiotoxicity, thus providing Auger electron-emitting radiotherapeutics with higher cell-specificity and reduced “cross-fire” damage to adjacent healthy tissue in comparison to long-range β-particles (range in tissue: 50 μm–1.0 cm).^12^ A clinical example of an Auger therapeutic is ^111^In-DTPA-hEGF (DTPA = diethylenetriaminepentaacetic acid, hEGF = human epidermal growth factor), a radiopharmaceutical with high potency towards breast cancer cells that overexpress EGFR that has undergone a phase I clinical trial for the treatment of EGFR-positive breast cancer.^13^–^15^ Auger emitters conjugated to nanoparticles have also been explored for cancer cell targeting applications.^16^–^23^ The single-photon emission computed tomography (SPECT) imaging capability of ^111^In provides a means of direct visualisation of nanoparticle accumulation *in vivo*, thereby indicating the potential of ^111^In-conjugates as theranostics.^22^,^23^

The biocompatible and biodegradable polymer PLGA (poly(lactic-*co*-glycolic acid)) has been employed to form nanoparticles for delivering a wide variety of drugs *in vitro* and in pre-clinical experiments.^24^–^29^ As drug delivery candidates, the anti-cancer properties of substitutionally inert ruthenium(ii) polypyridyl complexes (RPCs) have been gaining increasing interest as alternatives to platinum-based therapeutics or highly potent organics.^30^–^32^ In particular, RPCs that bind DNA by metallo-intercalation rapidly stall DNA replication fork progression, activating DNA damage checkpoints and preventing growth of highly proliferative cancer cells by cell-cycle arrest.^33^,^34^ One complex, Ru(phen)_2_(tpphz)^2+^ (phen = 1,10-phenanthroline, tpphz = tetrapyrido[3,2-*a*:2′,3′-*c*:3′′,2′′-*h*:2′′′,3′′′-*j*]phenazine), **Ru1**, demonstrates potency comparable to that of cisplatin towards oesophageal cancer cells but with reduced cytotoxicity towards normal cells.^34^ Importantly, treatment with **Ru1** results in enhanced cancer cell sensitivity to radiotoxic external beam IR, indicating RPCs that target DNA replication may also function as radiosensitizers.^33^,^34^ However, improving cancer cell specificity is highly desirable to maximise potential therapeutic outcomes and minimise damage to healthy cells. Accordingly, work has investigated numerous RPCs in nanoparticle or liposome formulations,^35^–^43^ where their chemical stability, tuneable hydrophobicity and attractive photophysical properties are advantageous properties for drug delivery applications. To combine the therapeutic effects of IR and the radiosensitizing properties of **Ru1**, in this study we utilise PLGA nanoparticles to co-deliver ^111^In-labelled hEGF and **Ru1** to oesophageal cancer cells that overexpress EGFR, demonstrating both a combinational effect and molecular targeting for this novel therapeutic combination.

## Results

### Nanoparticle preparation and characterisation

**Scheme 1:**
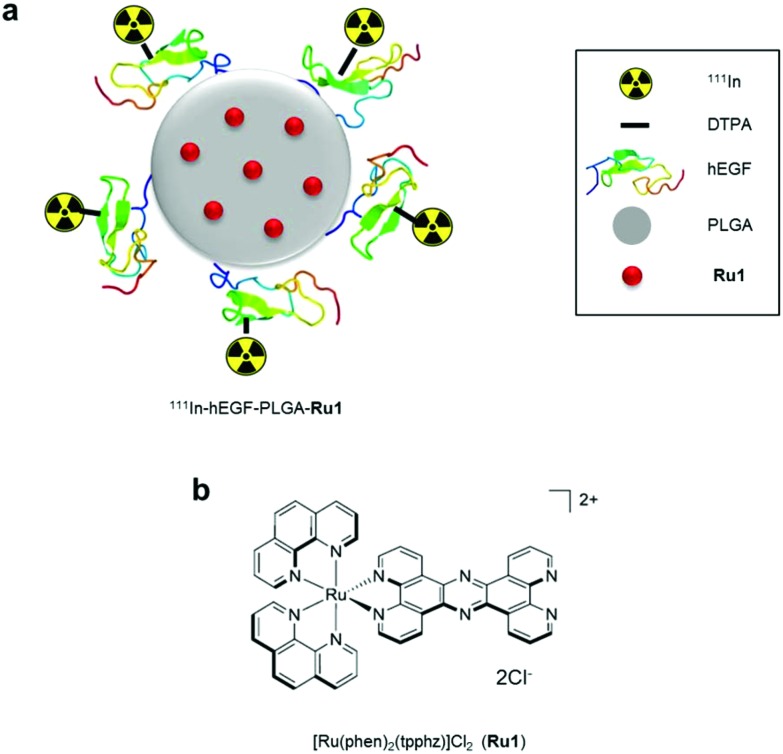
a) Radiolabelled nanoparticles employed in this study. The ruthenium(ii) metallo-intercalator and radiosensitizer, **Ru1**, is encapsulated within a PLGA core and nanoparticles are surface labelled with ^111^In-DTPA-hEGF. PLGA = poly(lactic-*co*-glycolic acid, **Ru1** = Ru(phen)_2_(tpphz)^2+^ (phen = 1,10-phenanthroline, tpphz = tetrapyrido[3,2-*a*:2′,3′-*c*:3′′,2′′-*h*:2′′′,3′′′-*j*]phenazine), hEGF = human epidermal growth factor, DTPA = diethylenetriaminepentaacetic acid. (b) Chemical structure of **Ru1**.

**Fig. 1:**
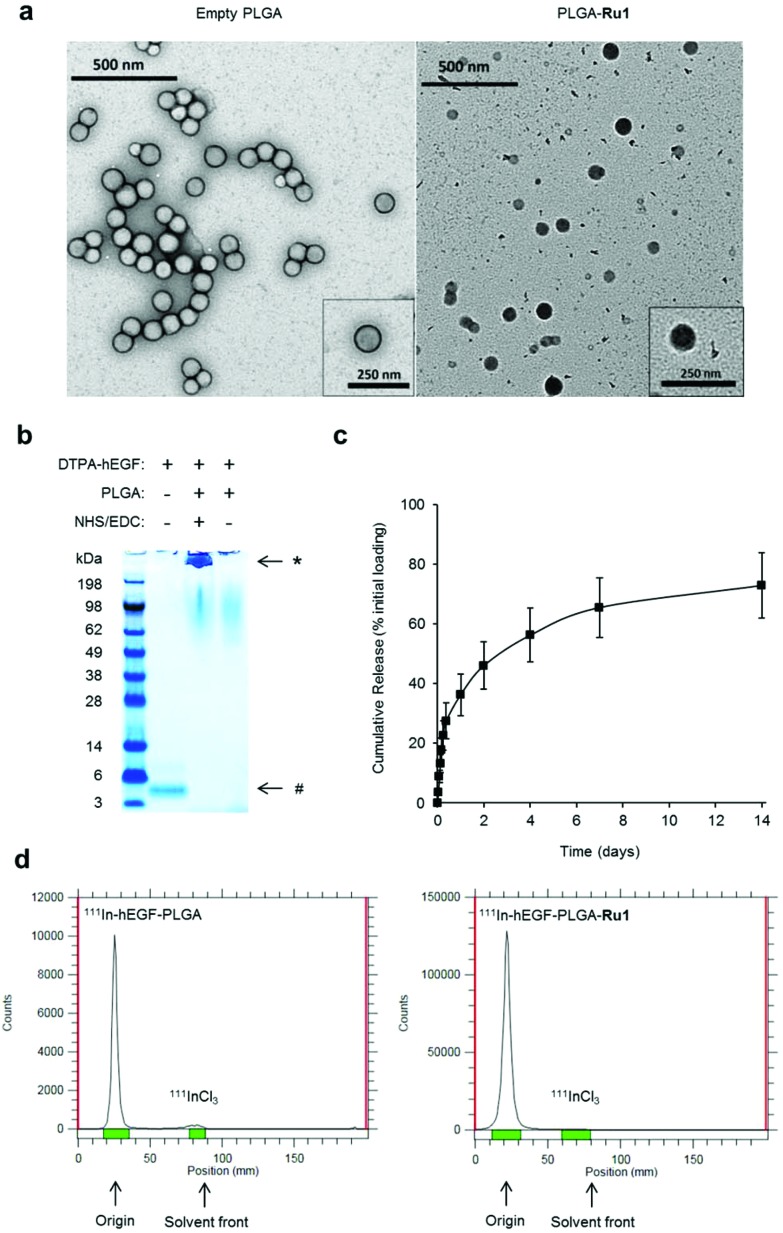
(a) TEM images of unloaded (left) and **Ru1**-containing (right) PLGA nanoparticles. For unloaded PLGA nanoparticles (left) uranyl acetate contrast stain was employed. For **Ru1**-loaded particles (right), no TEM contrast stain was used, thereby allowing direct visualisation of **Ru1** contrast within the PLGA core. (b) Coomassie blue stained SDS-PAGE gel of free hEGF (1 μg, left lane) or PLGA nanoparticles (1 mg) added to hEGF in the presence (middle lane) or absence (right lane) of NHS/EDC crosslinking agents. Nanoparticles were separated from unreacted hEGF by centrifugation before loading. *hEGF-PLGA, #hEGF. (c) Release kinetics of hEGF-PLGA-**Ru1** showing biphasic release profile of **Ru1**. (d) Representative instant thin layer chromatograms (iTLC) of purified ^111^In-hEGF-PLGA and ^111^In-hEGF-PLGA-**Ru1** nanoparticles in citrate buffer using EDTA (0.5 M, pH = 7.6) as the mobile phase.

PLGA nanoparticles encapsulating **Ru1** and surface conjugated to DTPA-hEGF were prepared (Scheme 1). This latter addition provides both the targeting ligand for EGFR and DTPA chelating groups for subsequent ^111^In-radiolabelling. A double emulsion evaporation method was used for the preparation of PLGA-**Ru1** nanoparticles, where a drug-to-polymer ratio of 1 : 6.25 resulted in 11.4 μg **Ru1** per mg nanoparticles; a drug loading content (L.C.%) of 1.14. Transmission electron microscopy (TEM) and dynamic light scattering (DLS) indicated nanoparticles were spherical and approximately 130–140 nm in diameter (Fig. 1a and Table 1). TEM also confirmed **Ru1**-loading within the PLGA core, as direct visualisation of the complex due to the electron-dense ruthenium is evident by this technique (Fig. 1a). DTPA-hEGF conjugated nanoparticles (hereafter referred to as hEGF-PLGA) were prepared by reacting PLGA nanoparticles with DTPA-hEGF employing established EDC/NHS (EDC = 1-ethyl-3-(3-dimethylaminopropyl)carbodiimide, NHS = *N*-hydroxysuccinimide) conjugation chemistry.^44^ This involves activation of the terminal carboxyl groups of PLGA through an NHS-PLGA intermediate before amide bond formation to DTPA-hEGF. A reaction ratio of 1 μg hEGF : 200 μg PLGA resulted in a final ratio of 2 μg hEGF per mg nanoparticles (Fig. S1 in the ESI†). Attachment of hEGF to PLGA was examined by SDS-PAGE. This technique relies on the principle that nanoparticles are unable to migrate through the gel whereas the denaturing conditions of SDS-PAGE breaks weak (non-covalent) bonds, thereby allowing separation of any unattached or adsorbed peptide. PLGA nanoparticles were washed before loading to remove unreacted hEGF. As shown in Fig. 1b, after electrophoretic separation, the addition of the protein stain Coomassie blue revealed a strong band at the top of the gel for PLGA reacted with hEGF in an NHS/EDC-dependent manner. The absence of a band corresponding to free hEGF protein at the bottom of the gel (molecular weight approximately 6.4 kDa) is consistent with the successful covalent conjugation of hEGF to PLGA and removal of unbound hEGF by washing. In the control sample which lacked NHS/EDC, no protein staining was seen at the top of the gel, confirming an absence of covalent attachment of hEGF to PLGA in this sample (Fig. 1b). The increase in nanoparticle zeta potential after reacting with hEGF (Table 1) is additionally consistent with successful surface-grafting of the peptide to PLGA.^44^ Examination of particle size by DLS over an extended period indicated nanoparticles were stable >30 days in solution, with no evidence of aggregation in this time frame (Table S1 and Fig. S2†). Encapsulated **Ru1** displayed a biphasic release profile involving release of 46% of the encapsulated **Ru1** in 2 days and slower release of a further 27% of the encapsulated compound in the subsequent 12 days (Fig. 1c). Radiolabelling of nanoparticles with ^111^InCl_3_ achieved a specific activity of 4–6 MBq ^111^In per mg hEGF-PLGA and a radiochemical purity of >95% after removal of unbound ^111^InCl_3_ (Fig. 1d). The absence of radiolabelling of native PLGA-**Ru1** particles confirmed DTPA-hEGF conjugation is required for successful radiolabelling (Fig. S3†).

**Table 1:** Physical properties of nanoparticles used in this study

Nanoparticles	Diameter (nm)	Polydispersity index	Zeta potential (mV)
PLGA	129.0	0.178	–22.8
PLGA-**Ru1**	138.8	0.087	–12.1
hEGF-PLGA	136.1	0.051	–3.4
hEGF-PLGA-**Ru1**	133.0	0.236	–4.7

### EGFR-mediated nanoparticle uptake

**Fig. 2:**
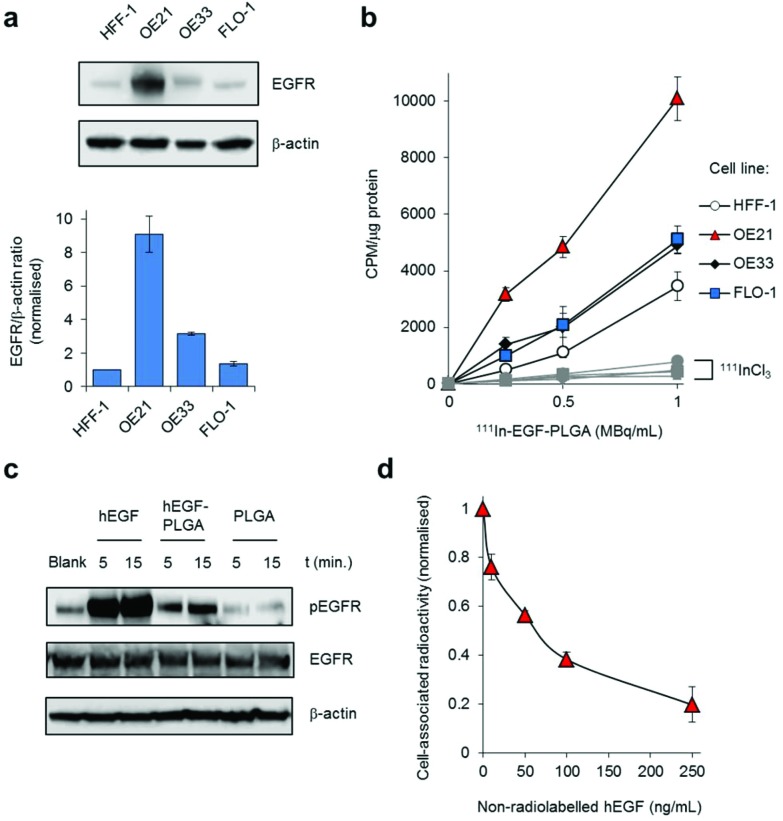
(a) EGFR levels in HFF-1 normal human fibroblasts and OE21, OE33 and FLO-1 oesophageal cancer cells, as determined by immunoblotting of whole-cell lysates with anti-EGFR antibodies (top) and quantified by densitometry (bottom). Data expressed as a ratio of EGFR to β-actin levels and are normalised to results for HFF-1 cells. Mean of two technical repeats ± S.D. (b) Uptake of ^111^In-labelled hEGF-PLGA nanoparticles by HFF-1, OE21, OE33 or FLO-1 cells, as assessed by internalised cellular radioactivity (2 h incubation). Data expressed as counts per minute (CPM) per μg cell protein. Mean of triplicates ± S.D. Results for each cell line treated with equivalent amounts of added radioactivity of ^111^InCl_3_ included for comparison (grey). (c) Western blot analysis of total EGFR and pEGFR (EGFR phosphorylated at Tyr1068) levels in OE21 cell lysates. Cells were serum-starved (24 h) before treatment with hEGF (50 ng mL^–1^), hEGF-PLGA (0.5 mg mL^–1^) or PLGA (0.5 mg mL^–1^) for 5 or 15 minutes. (d) Effect of EGFR blocking on ^111^In-hEGF-PLGA uptake in OE21 cells. Cells were pre-incubated with a concentration range of non-radiolabelled hEGF (1 h) before addition of ^111^In-hEGF-PLGA (0.5 MBq mL^–1^) plus non-radiolabelled hEGF (2 h co-incubation). The internalised radioactivity was measured and normalised to control (*i.e.* unblocked) conditions. Data are the mean of two independent experiments ± S.D., where each experiment was performed in triplicate.

The conjugation of hEGF aimed to achieve increased levels of nanoparticle uptake in oesophageal cancer cells that overexpress EGFR compared to cells with normal EGFR levels. First, immunoblotting and densitometry were used to confirm relative EGFR expression in three different oesophageal cancer cell lines. HFF-1 immortalised human foreskin fibroblasts were included as a non-cancer derived cell line. These results show the OE21 human oesophageal squamous cell carcinoma (ESCC) cell line expresses substantially greater levels of EGFR compared to either OE33 or FLO-1 human oesophageal adenocarcinoma cell lines (Fig. 2a). The number of EGFR receptors per cell for each cancer cell line was quantified by ^111^In-DTPA-hEGF competition binding assay and found to be 8.93 × 10^5^, 6.16 × 10^4^ and 4.76 × 10^3^ receptors per cell for OE21, OE33 and FLO-1 cells respectively. These EGFR expression results for the three cell lines are in agreement with publicly available data from the Cancer Cell Line Encyclopedia (CCLE).^45^ Next, the uptake of radiolabelled nanoparticles in each cell line was determined by quantifying total cell-associated radioactivity (internalised and surface-bound). This revealed EGFR-overexpressing OE21 cells demonstrated the greatest level of particle accumulation of the four cell lines tested; an approximately two-fold greater level of radioactivity than for either OE33 or FLO-1 cells (Fig. 2b and Table S2†). The lowest levels of internalisation were found in normal HFF-1 cells, correlating with these cells demonstrating the lowest EGFR expression (Fig. 2a and b). The similar level of nanoparticle uptake by OE33 and FLO-1 cells is consistent with their comparable EGFR expression. Compared to radiolabelled nanoparticle treatment, substantially decreased levels of radioactivity were observed in all four cell lines when treated with equivalent concentrations of free ^111^InCl_3_, confirming nanoparticle-mediated uptake was responsible for the majority of measured cellular radioactivity (Fig. 2b, grey).

hEGF binding to EGFR results in autophosphorylation of intracellular tyrosine residues of the receptor.^46^ Accordingly, the level of EGFR phosphorylated at one such autophosphorylation site, Tyr1068, was examined to provide an indication of successful EGF-EGFR binding. As shown in Fig. 2c, western blot analysis revealed that, compared to an untreated control sample, OE21 cells treated with hEGF-PLGA nanoparticles showed a rapid increase in the level of EGFR phosphorylated at Tyr1068 (pEGFR) with no change in total EGFR protein content. In contrast, there was no increase in pEGFR level above control in cells treated with non-hEGF conjugated PLGA nanoparticles (Fig. 2c). Finally, pre-blocking OE21 cells with non-radiolabelled hEGF before co-incubation of cells with ^111^In-labelled and hEGF-tagged particles resulted in a decrease in intracellular radioactivity with increasing hEGF concentration, where >80% of uptake was blocked at the highest concentration of hEGF employed (Fig. 2d). Together, these findings are consistent with (i) EGFR binding and (ii) EGFR-mediated cellular uptake of hEGF-PLGA nanoparticles.

### Subcellular distribution of ^111^In and Ru1

**Fig. 3:**
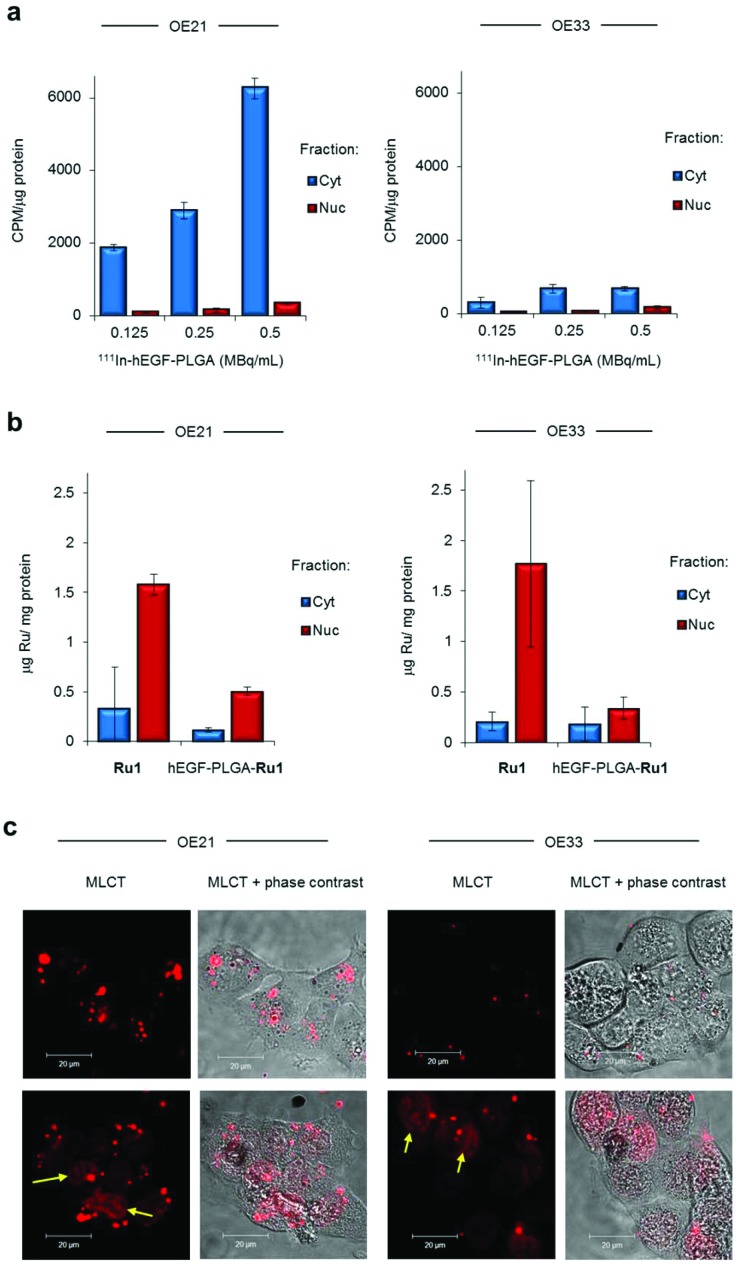
(a) Sub-cellular radioactivity content of OE21 or OE33 cells treated with ^111^In-hEGF-PLGA (0.125–0.5 MBq mL^–1^, 2 h). Isolated cytosol (Cyt) and nuclear (Nuc) fractions were obtained. The amount of accumulated radioactivity was measured by gamma-counting and normalised to protein content of each fraction (experiment performed in triplicate ± S.D.). See ESI† for verification of efficient sub-cellular fractionation and data expressed as % of total radioactivity added. (b) Sub-cellular ruthenium content of OE21 or OE33 cells treated with hEGF-PLGA-**Ru1** (1 mg mL^–1^, 24 h), as determined by ICP-MS. Data for cells treated with equivalent concentration of free **Ru1** (12 μM, 24 h) included for comparison. Data are normalised to protein concentration and are the mean of two independent experiments ± S.D. (c) Confocal microscopy (CLSM) of OE21 or OE33 cells treated with hEGF-PLGA-**Ru1** (1 mg mL^–1^, 24 h) showing intracellular MLCT (metal to ligand charge-transfer) emission of **Ru1**. Live cell imaging (top row) or the same cells visualised immediately after 4% formaldehyde fixation (bottom row). Identical imaging parameters were used for all images shown. Arrows indicate nuclear MLCT emission.

The short range of Auger electrons in biological media means cellular internalisation, and particularly nuclear uptake, is desirable to achieve radiotoxicity.^12^ On examining the subcellular distribution of internalised radioactivity in OE21 cells after treatment with ^111^In-hEGF-PLGA (2 h), ^111^In was found to have accumulated primarily in the cytosol with 5.1 ± 0.1% of the total cell-internalised radioactivity detected within the nuclear fractions (Fig. 3a and S4†). This subcellular distribution remained unchanged following exposure for up to 24 h (Fig. S5†). Similar subcellular distributions were obtained for OE33 cells treated with ^111^In-hEGF-PLGA, albeit at lower total cellular radioactivity due to reduced nanoparticle uptake in this cell line (Fig. 3a and S4†). In comparison to the results for hEGF-labelled nanoparticles, a greater level of total internalised radioactivity (14.8 ± 3.8%) was located within isolated nuclear fractions in cells treated with ^111^In-DTPA-hEGF peptide (Fig. S6†), in agreement with previous work and the nuclear translocation properties of EGFR.^13^,^47^

To assess **Ru1** uptake and localisation, ruthenium content of nanoparticle-treated cells was determined by inductively coupled plasma mass spectroscopy (ICP-MS). This indicated that the majority (>65%) of total intracellular Ru content was detected in isolated nuclear fractions of cells treated with **Ru1**-loaded nanoparticles after 24 h (Fig. 3b). These results additionally indicated Ru content in nanoparticle-treated cells was approximately 1.5-fold higher in OE21 cells compared to OE33; a result in agreement with radioactivity data above (Fig. 2b). Surprisingly, these results also indicated the amount of Ru detected was lower than cells treated with an equivalent concentration of free **Ru1**. This finding may be explained by relatively low loading of **Ru1** within PLGA, a common outcome for hydrophilic compounds,^24^ and also different uptake pathways: PLGA nanoparticles are thought to be internalised primarily by endocytosis^48^ while a non-endocytic mechanism of active transport has been indicated for **Ru1**.^49^ Finally, as **Ru1** is an metal to ligand charge transfer (MLCT) “light switch” complex that demonstrates a large increase in emission intensity when bound to DNA (ref. 49 and Fig. S7†), we examined nanoparticle-treated cells by confocal laser scanning microscopy. Applying this technique, luminescence in the cell cytosol was visible along with clear evidence of nuclear-localised **Ru1** (Fig. 3c and Fig. S8†). Taken together, these results show that the majority of the nanoparticles themselves remain in the cell cytosol while the greater levels of nuclear-targeting demonstrated by **Ru1** compared to ^111^In indicate the successful release of the complex from the nanoparticles.

### Nanoparticle impact on cell proliferation

**Fig. 4:**
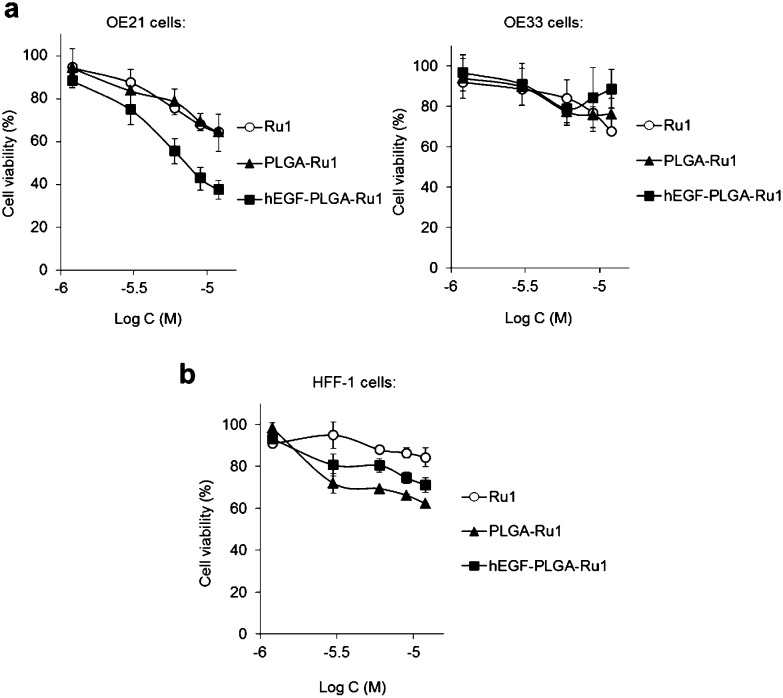
(a) Impact of **Ru1**-containing hEGF-PLGA or PLGA nanoparticles on cell viability of OE21 (overexpressed EGFR) or OE33 (normal EGFR levels) oesophageal cancer cells. Cell viabilities determined by MTT assay (24 h incubation) and expressed as a function of **Ru1** concentration. The equivalent concentration of free **Ru1** is included for comparison. (b) Impact of **Ru1**-containing nanoparticles on cell viability of HFF-1 normal human fibroblasts. Data for (a,b) are the mean of two or three independent experiments ± S.D, where each experiment was performed in triplicate.

Investigation of the impact of non-radiolabelled nanoparticles on the cell viability of OE21 or OE33 cells by MTT assay showed that **Ru1**-loaded hEGF-PLGA particles demonstrate relatively mild cytotoxic effects due to the low loading of **Ru1**. Despite this, it was evident that hEGF-PLGA particles showed greatest cytotoxicity in EGFR-overexpressing OE21 cells, where a greater impact on cell viability compared to either free complex or non-targeted PLGA-**Ru1** nanoparticles was achieved (Fig. 4a and Table S4†). In comparison to OE21 cells, hEGF-PLGA-**Ru1** had a reduced impact on EGFR-normal OE33 cells, in which cell viabilities >70% compared to untreated were obtained (Fig. 4a). In addition to these results employing oesophageal cancer cell lines, decreased potency of hEGF-PLGA-**Ru1** toward HFF-1 normal human fibroblasts compared to OE21 cells was seen, where cell viabilities remained at >70% compared to untreated cells (Fig. 4b and Table S4†). No obvious cytotoxicity of native (empty) PLGA nanoparticles was observed (Fig. S9†).

**Fig. 5:**
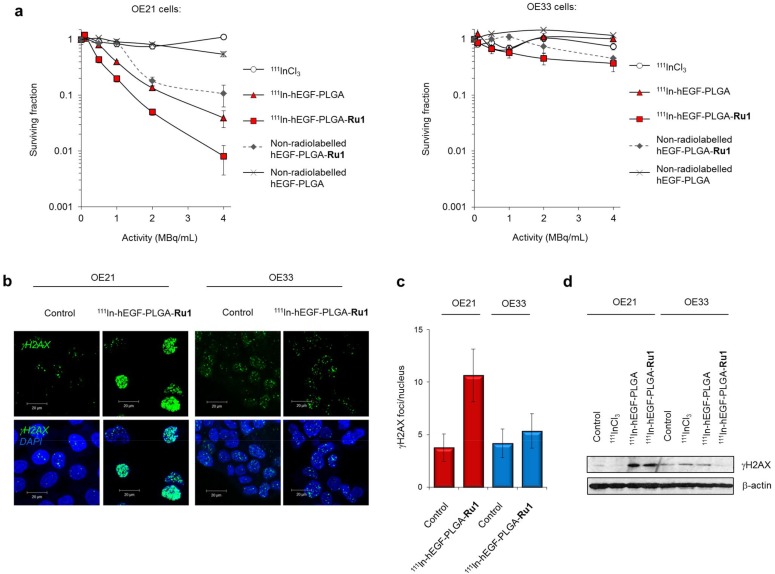
(a) Clonogenic survival assays of OE21 or OE33 cells exposed to ^111^In radiolabelled hEGF-PLGA nanoparticles with or without **Ru1**-loading (24 h incubation time). Non-radiolabelled nanoparticles (in equivalent concentrations; 0–1000 μg mL^–1^) and free ^111^InCl_3_ (in equivalent specific activity) are included as controls. Data points are the mean of triplicates ± S.D. (b) Representative CLSM images of OE21 or OE33 cells treated with ^111^In-hEGF-PLGA-**Ru1** (2 MBq mL^–1^, 24 h) followed by immunofluorescence staining for γH2AX (green). DNA was counterstained with DAPI (blue). See ESI† for full micrographs. (c) Quantification of γH2AX foci/nucleus (in plane of view) for cells treated as in (b). Data are the average of two independent experiments ± SD. A minimum of 100 nuclei were counted for each treatment group. (d) Immunoblotting of OE21 or OE33 whole-cell lysates for γH2AX expression after treatment with ^111^In-hEGF-PLGA or ^111^In-hEGF-PLGA-**Ru1** (2 MBq mL^–1^, 24 h). β-Actin was used as a loading control. Cells treated with free ^111^InCl_3_ (specific activity equivalent) were included for comparison.

Next, the effect of radiolabelled ^111^In-hEGF-PLGA nanoparticles on cell survival was measured by clonogenic survival assay; the standard method to assess radiotoxicity as this provides a long-term measure of proliferation inhibition. OE21 and OE33 cell lines were selected as representative EGFR overexpressing and EGFR normal cells, respectively. Cells were treated in parallel with equivalent concentrations of non-radiolabelled nanoparticles to evaluate the effect on cell survival of incorporation of ^111^In into the nanoparticle construct. These data indicated that treatment with ^111^In-labelled hEGF-PLGA nanoparticles induced a substantial decrease in clonogenic survival (surviving fractions, S.F., <0.2 relative to untreated) in OE21 cells at specific activities of 1 MBq mL^–1^ and greater (Fig. 5a). Treatment with free ^111^InCl_3_ did not impact cell proliferation up to the maximum concentration of 4 MBq mL^–1^ (Fig. 5a), consistent with the principle that cellular internalisation of ^111^In is a requirement for radiotoxicity.^13^ Notably, the inclusion of **Ru1** resulted in ^111^In-hEGF-PLGA-**Ru1** particles demonstrating two- to five-fold greater cytotoxicity than empty ^111^In-hEGF-PLGA (Fig. 5a and Table S5†). The highest concentration of empty hEGF-PLGA nanoparticles (1 mg mL^–1^) was found to have a minor impact on OE21 cell survival, likely due to the cytotoxic effects of hEGF peptide towards EGFR-overexpressing cells.^50^ By comparison to data for single-agent nanoparticle formulations hEGF-PLGA-**Ru1** and ^111^In-hEGF-PLGA, the overall impact of ^111^In-hEGF-PLGA-**Ru1** on OE21 cell survival indicates an additive contribution from both ^111^In surface-labelling and the inclusion of **Ru1** within the nanoparticles (Table S5 and Fig. S10†). ^111^In-hEGF-PLGA showed low radiotoxicity towards normal EGFR OE33 cells (S.F.s > 0.66) while ^111^In-hEGF-PLGA-**Ru1** nanoparticles were substantially less toxic than parallel results for OE21 cells, where an approximately 45-fold greater activity towards EGFR overexpressing cells at the highest dose of ^111^In-hEGF-PLGA-**Ru1** was observed (Fig. 5a and Table S5†). Due to their inability to form colonies, HFF-1 cells were incompatible with clonogenic survival assays, however, assessment of cell viability by MTT assay showed reduced radiotoxicity of radiolabelled nanoparticles towards HFF-1 normal fibroblasts compared to OE21 cells (Fig. S11†). These results are in agreement with the ^111^In-labelled hEGF-PLGA nanoparticles demonstrating increased radiotoxicity towards cells that overexpress EGFR compared to cells that express normal levels of the receptor and additionally show that cytotoxicity may be further enhanced by inclusion of **Ru1**.

### Nanoparticle-induced DNA damage

Auger electron radiotherapeutics are able to induce DNA damage if in close proximity to the cell nucleus^14^ while the radiosensitizing effects of **Ru1** for ^137^Cs γ-rays have been shown to occur through DNA damage enhancement.^34^ Accordingly, DNA damage generated as a result of radiolabelled nanoparticle exposure was examined. Treatment of cells with ^111^In-hEGF-PLGA-**Ru1** (2 MBq mL^–1^, 24 h) resulted in increased levels of the DNA damage marker γH2AX in OE21 cells compared to an untreated control sample (Fig. 5b and c). This result was supported by immunoblotting for γH2AX expression in lysates prepared from ^111^In-hEGF-PLGA- or ^111^In-hEGF-PLGA-**Ru1**-treated OE21 cells (Fig. 5d). A minimal increase in γH2AX levels was observed in EGFR normal OE33 cells treated with radiolabelled nanoparticles and also when either cell line was treated with free ^111^InCl_3_ (Fig. 5b–d); findings consistent with nanoparticle-mediated ^111^In uptake being responsible for the observed DNA damage.

**Fig. 6:**
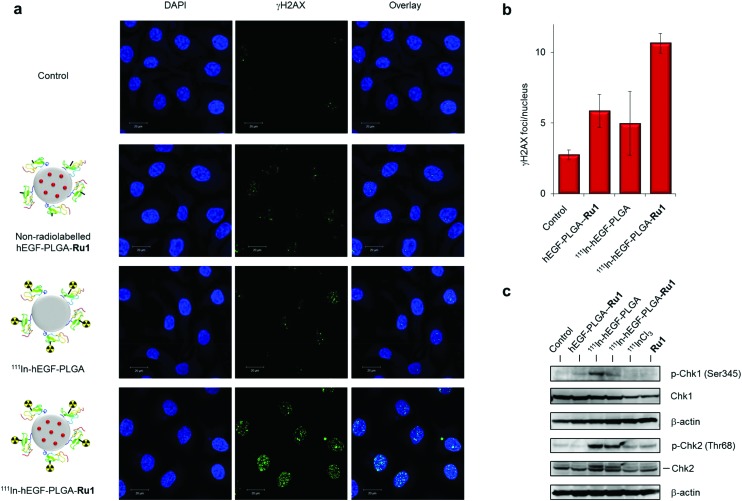
(a) CLSM images of OE21 cells treated with ^111^In-hEGF-PLGA or ^111^In-hEGF-PLGA-**Ru1** (1 MBq mL^–1^, 24 h) followed by immunofluorescence staining for γH2AX (green). DNA was stained with DAPI (blue). Equivalent non-radiolabelled hEGF-PLGA-**Ru1** treatment was included for comparison. (b) Quantification of γH2AX foci/nucleus (in plane of view) for cells treated as in (a). Data average of two independent repeats ± S.D. A minimum of 100 nuclei per condition were counted. (c) Immunoblotting of OE21 whole-cell extracts after 24 h treatment with non-radiolabelled hEGF-PLGA-**Ru1**, ^111^In-hEGF-PLGA or ^111^In-hEGF-PLGA-**Ru1** (1 MBq mL^–1^) using anti-pChk2 (Thr68) or pChk1 (Ser345) antibodies, as indicated. Total Chk2 and Chk1 protein content is provided. β-Actin was used as a loading control. Cells treated with free ^111^InCl_3_ (specific activity equivalent) or free **Ru1** (concentration equivalent) were included for comparison.

To assess the individual contributions of ^111^In and **Ru1** to DNA damage generation, single- or dual-agent nanoparticle formulations were prepared and levels of γH2AX foci in cells treated with equivalent doses were elucidated. These results indicated that the level of γH2AX foci is greatest for the nanoparticle formulation that contains both ^111^In and **Ru1** (Fig. 6a). Moreover, quantification of γH2AX foci/nucleus indicates DNA damage induced by ^111^In-hEGF-PLGA-**Ru1** equates to the sum of that caused by the individual single-agent nanoparticle treatment conditions, hEGF-PLGA-**Ru1** and ^111^In-hEGF-PLGA (Fig. 6b). In addition to increased γH2AX levels, treatment of OE21 cells with ^111^In-hEGF-PLGA or ^111^In-hEGF-PLGA-**Ru1** (1 MBq mL^–1^, 24 h) resulted in marked induction of phospho-Chk2 (Thr68), while the appearance of phospho-Chk1 (Ser345) was also observed (Fig. 6c). These cellular responses indicate that nanoparticle-conjugated ^111^In activates both double-strand break (DSB) damage and single-strand break (SSB) DNA damage response (DDR) signalling after successful cellular internalisation, in agreement with Auger electrons inducing both forms of DNA lesion.^14^ Together, these findings indicate that dual delivery of **Ru1** and ^111^In results in enhanced DNA damage in oesophageal cancer cells that overexpress EGFR and are consistent with an additive therapeutic relationship between **Ru1** and ^111^In.

### Nanoparticle biodistribution

One aspect of the proposed therapeutic use of nanoparticles is the ability of nanoparticles to target tumours *in vivo*. Gamma-radiation emitted by ^111^In may provide detailed biodistribution information to assess tumour accumulation of ^111^In-radiolabelled nanoparticles.^23^ Accordingly biodistribution of ^111^In-labelled hEGF-PLGA nanoparticles was examined. ^111^In-hEGF-PLGA or ^111^In-hEGF-PLGA-**Ru1** nanoparticles were administered to mice bearing oesophageal tumour xenografts by intravenous injection and the resultant biodistribution determined by quantifying radioactivity in organs. OE21 cells were used as a representative EGFR-overexpressing cell line for generation of xenografts. Following sub-cutaneous inoculation of OE33 cells in BALB/c Nu/Nu mice, xenograft tumours grew very slowly, consistent with the reported experience of others.^51^ FLO-1 cells were therefore used as a representative cell line with normal EGFR expression for generation of xenografts. At 24 h post-injection of ^111^In-hEGF-PLGA or ^111^In-hEGF-PLGA-**Ru1**, uptake of radioactivity into the liver, spleen and kidneys was prominent. Tumour uptake represented 0.6–1% of total injected dose per gram (I.D. per g) with a tumour-to-muscle (T/M) ratio in the range of 2.5–4 (Fig. S12 and S13†). There was no significant difference in the level of tumour-associated radioactivity or T/M ratios between OE21 and FLO-1 xenograft models (Fig. S12 and S13†).

## Discussion

Targeted combination therapy using functionalised nanoparticles with multiple therapeutic components and mechanisms-of-action may achieve high cell specificity and act to combat the common problem of drug-resistance.^10^,^52^ By developing radionuclide-labelled nanoparticles that encapsulate complementary small molecule(s), these principles can also be applied to the highly successful strategy of chemoradiotherapy with the substantial added benefit of cell-specific targeting. However, while utilising radiosensitizers alongside β-emitting radiopharmaceuticals has made appreciable progress in the clinic,^11^ combination therapy utilising Auger electron radiotherapeutics remains under-developed.

The potential for using nanoparticles to deliver Auger electron radiotherapy has been demonstrated in studies conducted by the Reilly and Allen groups.^16^–^20^ For example, block copolymer micelles of ∼30 nm diameter surface-labelled with trastuzumab (Tz) were used to target ^111^In to Her2-positive breast cancer cells.^20^ Although DNA damage was not investigated, these investigators showed that radiotoxicity could be enhanced by increasing nuclear targeting of ^111^In by addition of a nuclear localisation signal to Tz (with ∼8% of total internalised radioactivity accumulating in cell nuclei). Moreover, the inclusion of low dose methotrexate, a dihydrofolate reductase inhibitor and potent radiosensitiser, significantly enhanced cell killing. Similar to block copolymer micelles developed by Fonge *et al.*^19^ and our previous work employing ^111^In-hEGF-labelled gold nanoparticles,^21^,^22^ ^111^In-hEGF-PLGA nanoparticles described in the current study demonstrate substantial radiotoxicity towards p53-deficient EGFR-overexpressing cancer cells. Notably, radiotoxicity occurs with clear evidence of DNA damage induction, observable by strong activation of DDR signalling, despite the modest extent of nuclear localisation of ^111^In.

The discovery that RPCs may function as radiosensitizers for cancer cells has extended their potential therapeutic application to include combination with IR. Although RPCs have been examined as photosensitizers in nanoparticle formulation,^53^,^54^ the requirement for simultaneous light exposure and efficient delivery of nanoparticles to target cells for effective phototoxicity presents challenges. Given the restricted penetration of light in tissue, and limited options for localised light delivery at present, this approach is only suitable for superficial tumours. In contrast, a nanoparticle that combines a radiosensitizer and radionuclide can achieve concomitant co-delivery to a specified cell population in a primary tumour, distant metastases or both. To validate the dual delivery concept at the cellular level, the DNA replication inhibitor **Ru1** – the most potent RPC radiosensitizer described to date – plus ^111^In were included in dual-agent hEGF-PLGA targeted nanoparticles. This agent resulted in an additive increase in cytotoxicity in oesophageal cancer cells that overexpress EGFR. The additive increase in DNA damage with the inclusion of ^111^In and **Ru1** indicates a complimentary relationship between the two DNA-damaging components. These data are the first demonstration of an RPC and radionuclide being employed together in such a manner. Slow-release PLGA nanoparticles delivered a modest level of intracellular **Ru1** but was nonetheless efficacious, illustrating the potential of combining a radiosensitizer at low dose with high linear energy transfer (LET) Auger-emitting radionuclides *via* targeted nanoparticle delivery. The substantially reduced impact of ^111^In-hEGF-PLGA-**Ru1** on EGFR normal cells represents a greater degree of cell-specificity than obtained for free **Ru1** in the same cell lines^34^ and indicates how this nanoparticle formulation may achieve molecular targeting as well as concomitant co-delivery.

The synthetic preparation is ‘modular’ such that every component – loaded drug, targeting peptide and radionuclide – may be varied to account for the molecular characteristics of a specific cancer. A key strength of this approach is that the radionuclide labelling is the final step, minimising loss during synthesis due to decay. However, as indicated in preliminary studies presented here, relatively low accumulation of nanoparticles themselves is a common finding in solid tumours *in vivo* after intravenous injection.^55^ This may be seen in seminal work by Farokhzad and colleagues, where only 0.83% I.D. (injected dose) of PSMA-targeted PLGA nanoparticles localised to tumours in murine models.^27^ Indeed, a recent meta-analysis of over 270 publications demonstrated that the “enhanced permeability and retention (EPR)” effect provides an average passive accumulation level of 0.7% I.D. per g.^55^ Biodistribution studies have also indicated that high liver and spleen accumulation is a consequence of administering nanoparticles of this shape and size by the intravenous route.^55^,^56^ Despite this low tumour uptake, formulations based on polymeric nanoparticles that incorporate docetaxel have progressed to phase I and II clinical trials for patients with metastatic prostate cancer^57^,^58^ and several polymer-based nanoparticles are FDA-approved.^59^ It is also notable that the primary effect of adding targeting ligands on nanoparticles may well be to improve *cellular* internalisation rather than overall *tumour* accumulation.^10^ This may be evidenced in studies using ^111^In-labelled PSMA-targeted PLA (polylactic acid) nanoparticles where only a modest increase in tumour I.D. per g was found for targeted nanoparticles compared to non-targeted analogues.^23^ These observations support our strategy of using short-range Auger electron-emitting radionuclides, as their cellular uptake requirements for radiotoxicity means non-specific organ toxicity will be better controlled compared to long-range β particles. Finally, the combination is unlikely to be restricted to PLGA nanoparticles and recent advances in nanoparticle design^56^ or stimuli-based release mechanisms^60^ may further extend the combination of Auger electron-emitting radiopharmaceuticals and DNA-targeting radiosensitizers. Future work will aim to expand on these concepts.

## Experimental

### Chemicals

**Ru1** was prepared using methods described by Bolger, *et al.*^61^ All NMR, mass spectroscopy and elemental analysis were in agreement with published data. Unless stated otherwise, all other chemicals were obtained from Sigma. Antibodies: p-Chk1 (Ser345) and p-Chk2 (Thr68) (Cell Signaling), γH2AX (Millipore), total Chk1 (Santa Cruz), total Chk2, α-tubulin (both Abcam) and β-actin (Sigma). AlexaFluor488-conjugated anti-mouse secondary antibodies were from Cell Signaling. DTPA-hEGF was prepared as reported previously.^62^

### Nanoparticle preparation

PLGA nanoparticles were prepared by a standard procedure.^63^ For preparation of PLGA-**Ru1** nanoparticles, a double emulsion evaporation method was employed where aqueous **Ru1** (2.4 mg of **Ru1** in 200 μL DI water) was added to 0.75% w/v PLGA solution in chloroform. Following sonication for 1 min, this emulsion was added dropwise to 4 mL of 5% w/v polyvinyl alcohol (PVA). This suspension was then sonicated for 3 min (VibraCell VCX130, 60% amplitude, three cycles of 55 s on, 5 s off over ice) and stirred overnight. PLGA-**Ru1** or PLGA particles were collected by centrifugation (25 000 rpm for 40 min at 4 °C). For protein conjugation, particles (10 mg) were activated with NHS (*N*-hydroxysuccinimide, 60 mg) and EDC (ethyl(dimethylaminopropyl) carbodiimide, 72 mg) in MES buffer (0.1 M, pH 4.9) with shaking for 30 minutes at room temperature before the addition of hEGF-DTPA (20 μg) overnight. Conjugated nanoparticles were separated from unreacted DTPA-hEGF by centrifugation at 25 000 rpm for 40 minutes at 4 °C using a Beckman Coulter optima max ultracentrifuge. The amount of DTPA-hEGF present on the particles was determined using a Human EGF Quantikine ELISA kit (R&D Systems, Inc., Minneapolis, MN, USA) following the manufacturer's instructions.

### Nanoparticle characterisation

The relative hydrodynamic diameter of nanoparticles was determined by dynamic light scattering (DLS) using a Zetasizer Nano ZS instrument (Malvern Instruments). Zeta potential measurements were carried out using a disposable folded capillary Zeta cell filled with the nanoparticle suspension in deionised water at 20 °C according to the manufacturer's instructions. Zeta potential was calculated from the electrophoretic mobility of nanoparticles using the Smoluchowski approximation. For TEM, samples were prepared by air-drying 0.01 mg mL^–1^ nanoparticle solution on a 200-mesh Formvar-coated copper grid (Agar Scientific, Stansted, Essex, UK). Empty PLGA nanoparticles were negatively stained with 2% uranyl acetate. Nanoparticles were visualised on an FEI Tecnai T12 electron microscope. Drug loading efficiency was determined by absorption spectroscopy at 450 nm with reference to a standard curve of free drug absorbance/concentration. The release of **Ru1** from PLGA was evaluated by dispersing the particles (1 mg mL^–1^) in PBS and incubating them at 37 °C. At predetermined timepoints, the particles were centrifuged using a 10 000 MWCO filter for 10 min at 14 000 rpm. The supernatant was collected for analysis and the particles were re-dispersed in PBS before incubating them at 37 °C. Absorbance readings of the supernatant were performed at 450 nm to determine **Ru1** concentration. Excitation/emission spectra of **Ru1**-loaded nanoparticles was recorded by TECAN Infinite 200 PRO plate reader (excitation wavelength = 488 nm, emission wavelength = 630 nm). To confirm DTPA-hEGF conjugation, purified conjugated nanoparticles were loaded on NuPAGE® 4–12% Bis-Tris gels (1–2 mg nanoparticle per well) alongside free DTPA-hEGF protein (1–5 μg per lane). Gels were run in MES/TRIS/SDS running buffer, washed in water before staining with SimplyBlue™ SafeStain (Thermo) overnight. Gels were washed in water for 1–3 h to decrease background before visualisation on an HP ScanJet 5590 Digital Flatbed Scanner.

### Radiolabelling

^111^In labelled hEGF-PLGA nanoparticles were prepared by mixing ^111^InCl_3_ in 0.1 M sodium citrate buffer (pH = 5.5) with hEGF-PLGA or hEGF-PLGA-**Ru1** at 4 °C for 1 h. A ratio of 10 MBq ^111^In per mg nanoparticles (equivalent to 10 MBq per μg surface-conjugated hEGF) achieved approximately 40–60% nanoparticle-bound ^111^In, corresponding to a specific activity of 4–6 MBq ^111^In per mg hEGF-PLGA. Radiolabelled nanoparticles were separated from unbound ^111^In by centrifugation (13 000 rpm, 30 min) and the pellet resuspended in PBS before use. Radiochemical purity was evaluated using instant thin layer chromatography (iTLC, Eckert & Ziegler AR-2000 radio-TLC Imaging Scanner) on glass microfiber chromatography paper silicic acid with 0.5 M EDTA (pH = 7.6) as the mobile phase. Radiochemical purity of >95% was required for all experiments.

### Cell lines

OE21 human oesophageal squamous cell carcinoma, OE33 human adenocarcinoma cells and FLO-1 distal oesophageal adenocarcinoma cells were a generous gift from E. Hammond, Oxford University. OE21 and OE33 cells were cultured in RPMI cell media supplemented with 10% FBS and penicillin/streptomycin. FLO-1 were cultured in DMEM supplemented with 10% FBS and penicillin/streptomycin. HFF-1 cells were cultured in DMEM supplemented with 15% FBS and penicillin/streptomycin. Cell lines were maintained at 37 °C in an atmosphere of 5% CO_2_ and sub-cultured by trypsin. Cell lines were used at passage numbers 30 or lower and checked to be mycoplasma-free on a monthly basis. Stock solutions of **Ru1** as the dichloride salt (2 mM) were prepared in PBS before dilution in cell media. Cells treated with **Ru1** were shielded from light to minimise phototoxicity.

### EGF receptor density

OE21, OE33 and FLO-1 cells were seeded at 5000 cells per well and incubated overnight at 37 °C. The cells were then incubated with 8 nM of ^111^In-DTPA-hEGF with varying concentrations of unlabelled hEGF (0 to 1000 nM) to block EGFR and measure non-specific binding. Following incubation for 2 h, the cells were washed twice with PBS and treated with 0.1 M NaOH. Lysates were transferred to counting tubes for measurement of radioactivity using the WIZARD-2 Automatic Gamma counter (PerkinElmer). Cell protein was determined by BCA assay to calculate the specific binding of radioactivity per mg of cell protein. IC_50_ values were determined by GraphPad Prism (GraphPad, SanDiego, CA) and used to calculate the maximum number of receptors per cell (*B*_max_).

### Cellular uptake studies

For total cellular uptake of radiolabelled nanoparticles, cells were seeded in well plates and allowed to proliferate for 24 h. Cells were incubated with a concentration-gradient of radiolabelled nanoparticles, the radioactive cell media removed, and cells washed twice with PBS before being lysed in radioimmunoprecipitation assay (RIPA) buffer. Lysates were collected by pipette and radioactivity measured by WIZARD-2 Automatic Gamma Counter. Readings were normalised to the protein content for each sample, as determined by BCA assay. For subcellular fractionation, the Nuclei EZ Lysis kit (Sigma) was employed using a protocol adapted from Hoang *et al.*^20^ Briefly, OE21 or OE33 cells were treated in 12 well plates, washed with cold PBS (2 × 2 mL) before washing with acidified PBS (pH 2.5) to remove the surface-bound fraction. Next, 0.4 mL EZ lysis buffer was added, cells were detached by scraping, collected into eppendorf tubes, vortexed briefly and left for 5 minutes on ice. Samples were centrifuged (500 g, 5 min) and the supernatant (cytosol fraction) aspirated. The pellet (nuclear fraction) was re-suspended in 200 μl RIPA buffer. Successful fractionation of the two subcellular compartments was verified by immunoblotting using anti-α-tubulin (Sigma) and anti-histone H2AZ (Abcam) for cytosol and nuclear fractions, respectively (Fig. S5b†). Radioactivity was measured using a WIZARD-2 Automatic Gamma Counter. For ruthenium content, OE21 or OE33 cells were treated in 12 well plates, washed twice with cold PBS (2 mL) and nuclear and cytosol fractions obtained using the Nuclei EZ Lysis kit (Sigma), as described above. ICP-MS analysis was carried out as previously reported.^33^ Counts per minute or Ru content of isolated fractions were divided by protein content, as determined by BCA assay.

### MTT assay

Cells were seeded in 96 well plates (10 000 cells per well) and allowed to adhere for 24 h. Cells were treated with 0–1000 μg mL^–1^ nanoparticle solutions (or the equivalent concentration of free **Ru1**) for 24 h. After incubation, 0.5 mg mL^–1^ MTT (thiazolyl blue tetrazolium bromide) dissolved in serum-free medium was added for 60 minutes and the formazan product eluted using acidified isopropanol. Absorbance at 540 nm was quantified by plate reader (reference wavelength 650 nm) and the metabolic activity of cell populations was determined as a percentage of a negative (solvent) control.

### Clonogenic survival assay

OE21 or OE33 cells were seeded in 12 well plates, allowed to adhere for 24 h and incubated with unlabelled or radiolabelled Na_2_VO_4_ nanoparticles for 24 h. Cells were detached using trypsin and re-seeded in 6 well plates at a density of 300–2000 cells per well (in triplicate). Cells were incubated for 7–14 days after re-seeding to allow colony formation before being fixed with 10% methanol, 10% acetic acid and stained with 0.4% methylene blue. OE33 cells required an additional formaldehyde fixation (4% in PBS, 5 min) step before staining to prevent colony detachment. Colonies containing 50 cells or greater were counted using a Gelcount instrument and accompanying software (Oxford Optronix). Plating efficiencies were determined for each treatment condition and normalised to an untreated control to provide the survival fraction (S. F.).

### Microscopy and immunofluorescence

Cells were seeded on Ibidi 35 mm μ-dishes (Thistle Scientific) and allowed to adhere for 24 h before treatment. Live cell imaging conditions maintained cells at 37 °C and 5% CO_2_ for the duration of the experiment. For fixed cell imaging, cell media was removed, cells washed with PBS and fixed with formaldehyde (4%, 10 min). For immunofluorescence, cells were permeablised with Triton (0.5% in PBS, 5 min) and washed with PBS. Samples were blocked with BSA (3% in PBS-T) for 1 h before incubation with primary antibody (γH2AX, 1/250 dilution) in BSA (3% in PBS-T) for 1 h. Samples were washed 3 times in PBS-T and incubated with AlexaFluor488-conjugated anti-mouse secondary antibodies (3% BSA in PBS-T, 1 h, 1/250 dilution). After washing three times (5 min PBS-T), samples were co-stained with DAPI (5 ng mL^–1^, 2 min) and fresh PBS added. Samples were visualised using a Zeiss LSM 780 inverted confocal microscope and an EC Plan-Neofluar 40×/1.30 Oil objective. DAPI, AlexaFluor488 and **Ru1** emission signals were collected as reported in a recent publication.^34^ γH2AX foci were counted using ImageJ software and the number of nuclei counted manually.

### Western blotting

After treatment, samples were washed with cold PBS and lysed in RIPA buffer containing protease inhibitors (10 μg mL^–1^ leupeptin, 2 μg mL^–1^ pepstatin, 50 μg mL^–1^ antipain, 2 μg mL^–1^ aprotinin, 20 μg mL^–1^ chyprostatin, 2 μg mL^–1^ benzamidine, 1 mM phenylmethanesulfonyl fluoride) and phosphatase inhibitors (50 mM NaF, 1 mM Na_3_VO_4_ and 20 mM β-glycerophosphate). Protein content was determined by BCA assay. Aliquots of cell lysates (10–50 μg total protein) were prepared in standard Laemmli buffer, heated at 95 °C for 5 minutes and resolved by NuPAGE® 4–12% Bis-Tris gels and LDS-PAGE. Gels were transferred onto nitrocellulose membrane and probed with primary antibodies in 5% BSA (bovine serum albumin). Reactions were visualised with a suitable secondary antibody conjugated with horseradish peroxidase (1/5000 dilution, Thermo). Pierce ECL (Thermo) or WesternSurePREMIUM (Li-Cor) chemiluminescent substrates with X-ray development (Fuji medical film and Optimax 2010 processor) or digital analysis (LiCor C-Digit Blot Scanner) were used to visualise protein expression.

### Animal models

Animal procedures were carried out in accordance with the UK Animals (Scientific Procedures) Act 1986 and with local ethical committee approval. OE21 or FLO1 tumours were established by subcutaneous injection of 2 × 10^6^ cells suspended in 200 μL 1 : 1 RPMI : Matrigel into the right dorsal flank of female BALB/c Nu/Nu mice. When tumours reached a size of approximately 8 mm geometric mean diameter (GMD), mice were assigned randomly into treatment groups (3 or 4 mice per group) and ^111^In-labelled nanoparticles (0.1 mg, 0.4 MBq, 5 mg kg^–1^) were administered by intravenous injection. At 24 h post injection (p.i.), mice were euthanised and organs were removed, weighed, and counted for radioactivity using a WIZARD-2 Automatic Gamma Counter.

## Conclusions

In summary, we demonstrate hEGF-PLGA nanoparticles can be utilised to combine the therapeutic effects of an Auger electron-emitting radionuclide, the controlled release of a radiosensitizing small molecule and cell receptor targeting. By using ^111^In-DTPA-hEGF and the ruthenium-based replication inhibitor [Ru(phen)_2_(tpphz)]^2+^ (**Ru1**), we show how co-delivery results in a substantially greater decrease in cell survival in EGFR-overexpressing oesophageal cancer cells compared to cells with normal EGFR expression. Finally, by comparison to single-agent formulations, we demonstrate this outcome is the result of an additive increase of DNA damage generated by ^111^In and **Ru1**, thereby verifying the nanoparticle-mediated therapeutic combination of an Auger electron emitter and DNA-targeting radiosensitizer.

## Conflicts of interest

There are no conflicts of interest to declare.

## Supplementary Material

Supplementary information
