# Supplementary material for: 111In-labelled polymeric nanoparticles incorporating a ruthenium-based radiosensitizer for EGFR-targeted combination therapy in oesophageal cancer cells
Source: Nanoscale. 2018 May 29;10(22):10596–608. doi: 10.1039/c7nr09606b (PMC5994990; doi:10.1039/c7nr09606b)
Supplement: Supplementary file 1 [file NR-010-C7NR09606B-s001.pdf]

Electronic Supplementary Material (ESI) for Nanoscale.
This journal is © The Royal Society of Chemistry 2018

Supplementary information for:

^{111}In-labelled polymeric nanoparticles incorporating a ruthenium-based radiosensitizer for EGFR-targeted combination therapy in oesophageal cancer cells†

Martin R. Gill,[a]‡ Jyothi U. Menon,[a,b]‡ Paul J. Jarman,[c] Joshua Owen,[b] Irini Skaripa-Koukelli,[a,b] Sarah Able,[a] Jim A. Thomas,[c] Robert Carlisle,[b] Katherine A. Vallis[a]*

a CRUK/MRC Oxford Institute for Radiation Oncology, Department of Oncology, University of Oxford, Oxford, UK. email: Katherine.vallis@oncology.ox.ac.uk

b Institute of Biomedical Engineering, Department of Engineering Science, University of Oxford, Old Road Campus Research Building, Oxford OX3 7DQ, UK.

c Department of Chemistry, University of Sheffield, Sheffield, UK.

Supplementary Tables

Table S1 Size and polydispersity index of hEGF-PLGA nanoparticles measured by DLS over an extended time period. Nanoparticles were stored in DI H_2O at room temperature. Mean of three readings +/- S.D.

Day	Diameter (nm)	S.D.	Polydispersity index	S.D.
0	136.1	2.1	0.051	0.018
1	134.3	2.4	0.078	0.017
4	133.8	1.0	0.050	0.018
6	135.1	2.7	0.040	0.013
8	136.6	2.4	0.064	0.016
11	134.3	2.0	0.072	0.005
13	133.8	1.6	0.062	0.022
15	135.4	2.5	0.050	0.040
18	135.1	2.8	0.065	0.022
20	132.4	1.3	0.059	0.022
25	135.4	2.2	0.059	0.013
30	133.5	1.5	0.047	0.017

Table S2. Cellular radioactivity of OE21, OE33, FLO-1 oesophageal cancer or HFF-1 normal fibroblast cells after treatment with ^{111}In-labelled hEGF-PLGA nanoparticles (2 h incubation at stated amount of radioactivity). Data expressed as counts per minute (CPM) per μg cell protein, as determined by BCA assay. Mean of triplicates +/- S.D.

	OE21		OE33		FLO-1		HFF-1	
MBq/mL	CPM/μg protein	SD	CPM/μg protein	SD	CPM/μg protein	SD	CPM/μg protein	SD
0	0.15	0.10	0.58	0.14	0.16	0.06	2.69	0.68
0.25	3173.34	237.80	1387.08	247.74	984.00	102.73	500.57	39.84
0.5	4835.02	370.76	2006.57	727.42	2074.50	417.21	1106.26	168.01
1	10089.17	765.11	4887.88	262.23	5097.93	490.63	3453.12	503.02

Table S3. Cellular radioactivity of OE21, OE33, FLO-1 oesophageal cancer or HFF-1 normal fibroblast cells after treatment with $^{111}InCl_3$ (2 h incubation at stated amount of radioactivity). Data expressed as counts per minute (CPM) per μg cell protein, as determined by BCA assay. Mean of triplicates +/- S.D.

	OE21		OE33		FLO-1		HFF-1	
MBq/mL	CPM/μg protein	SD	CPM/μg protein	SD	CPM/μg protein	SD	CPM/μg protein	SD
0	0.61	0.40	0.65	0.63	0.44	0.19	7.71	2.94
0.25	114.99	27.00	108.32	11.66	116.48	49.35	181.42	12.40
0.5	247.33	146.59	158.49	9.51	315.57	53.93	351.82	17.38
1	268.11	89.74	480.98	214.02	433.18	83.68	792.75	66.31

Table S4. Derived half-inhibitory (IC_{50}) concentrations for PLGA-**Ru1** and hEGF-PLGA-**Ru1** nanoparticles towards OE21 oesophageal squamous cell carcinoma, OE33 oesophageal adenocarcinoma or HFF-1 human foreskin fibroblast cells (24 h incubation, 0-1000 μg/mL nanoparticles, IC_{50} values expressed as μM **Ru1** where loading = 11.4 μg **Ru1**/mg nanoparticles). Data average of two independent experiments. [a]Data from Reference 1 included for comparison.

	OE21	OE33	HFF-1
**Ru1**	21 +/- 4.2[a]	44.5 +/- 3.5[a]	>100
PLGA-**Ru1**	>12	>12	>12
hEGF-PLGA-**Ru1**	7.5 +/- 2	>12	>12

Table S5. Surviving fraction (S.F.) of OE21 or OE33 oesophageal cancer cells treated with hEGF-PLGA-**Ru1,** ^{111}In-hEGF-PLGA, or ^{111}In-hEGF-PLGA-**Ru1** at the stated concentration for 24 h. Cell surviving fractions (S. F.) were determined by clonogenic survival assay and are the mean +/− S.D. of triplicates. Data were normalised to a untreated control for each experiment. OE33/OE21 S.F. ratio provides an indication of selectivity towards EGFR-overexpressing (OE21) versus EGFR normal (OE33) cells.

Treatment	OE21 cells		OE33 cells		OE33/OE21 S.F. Ratio
hEGF-PLGA-**Ru1** (mg/mL)	S.F.	S.D.	S.F.	S.D.	
0	1.000	0.008	1.000	0.169	**1.0**
0.125	0.854	0.064	1.000	0.184	**1.2**
0.25	0.875	0.142	1.095	0.124	**1.3**
0.5	0.180	0.032	0.736	0.170	**4.1**
1	0.106	0.045	0.454	0.026	**4.3**
^{111}In-hEGF-PLGA (MBq/mL)	S.F.	S.D.	S.F.	S.D.	
0	1.000	0.018	1.000	0.280	**1.0**
0.5	0.795	0.026	0.707	0.112	**0.9**
1	0.395	0.024	0.660	0.036	**1.7**
2	0.135	0.010	1.089	0.170	**8.1**
4	0.039	0.013	1.017	0.078	**26.1**
^{111}In-hEGF-PLGA-**Ru1** (MBq/mL)	S.F.	S.D.	S.F.	S.D.	
0	1.000	0.051	1.000	0.135	**1.0**
0.5	0.434	0.053	0.676	0.124	**1.6**
1	0.199	0.025	0.577	0.119	**2.9**
2	0.050	0.006	0.453	0.104	**9.1**
4	0.008	0.004	0.371	0.109	**46.4**

Supplementary Schemes and Figures

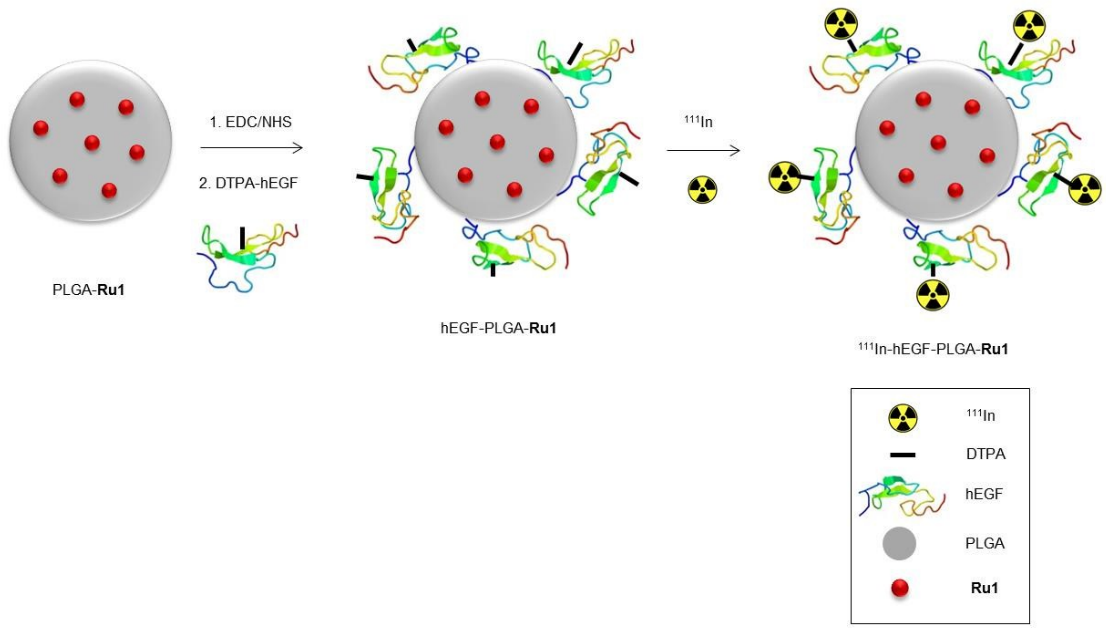

Scheme S1 Preparation of radiolabelled nanoparticles employed in this study. PLGA = poly(lactic-co-glycolic acid, **Ru1** = Ru(phen)$_2$(tpphz)$^{2+}$ (phen = 1,10-phenanthroline, tpphz = tetrapyrido[3,2-a:2′,3′-c:3′′,2′′-h:2′′′,3′′′-j]phenazine), hEGF = human epidermal growth factor, DTPA = diethylenetriaminepentaacetic acid.

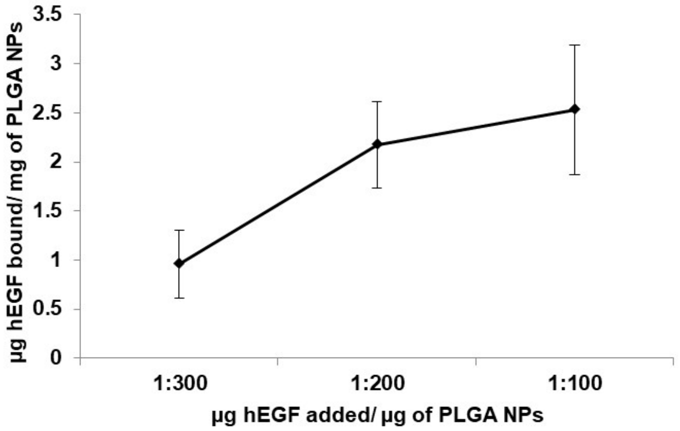

Figure S1 hEGF concentration on the surface of PLGA nanoparticles prepared from different hEGF:PLGA reaction ratios. hEGF concentration determined by ELISA assay.

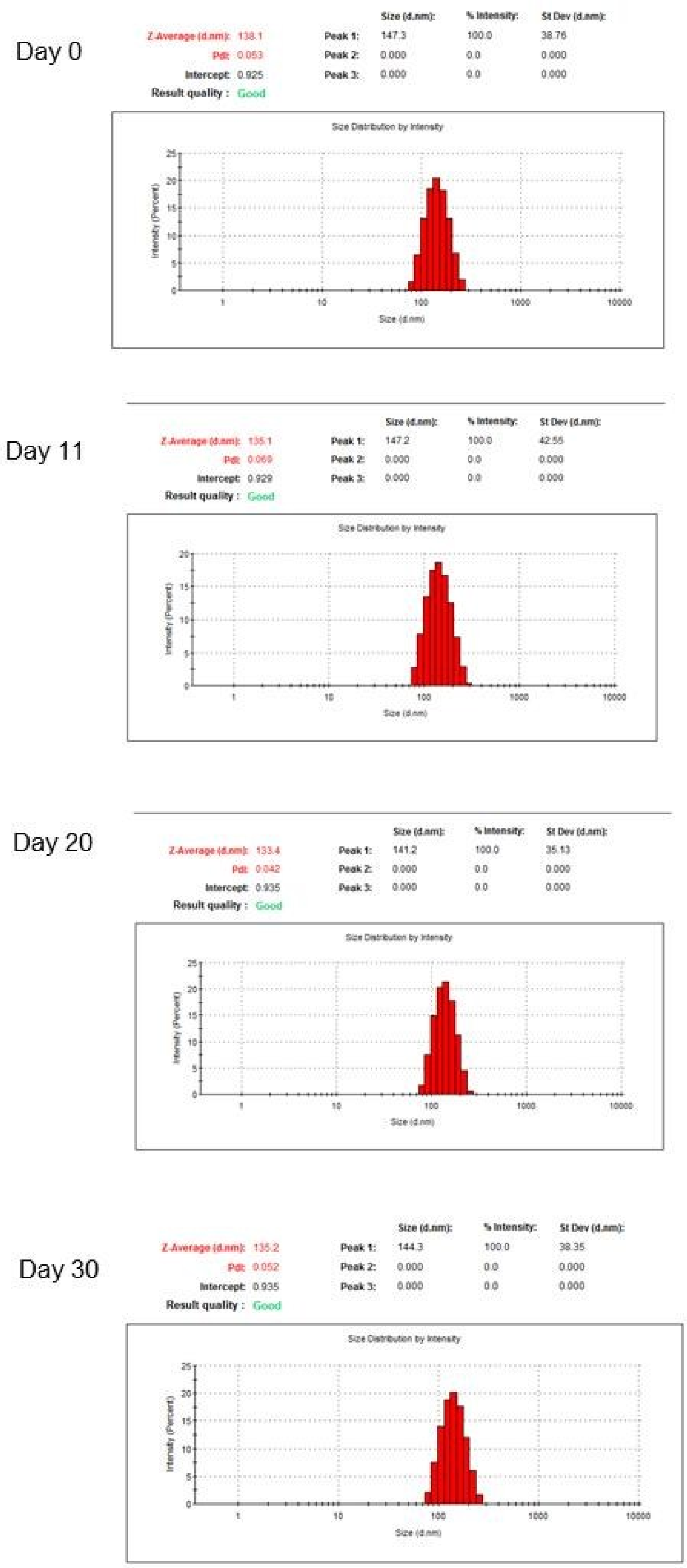

Figure S2 Examples of DLS of hEGF-PLGA nanoparticles over an extended time period. Nanoparticles were stored in deionised water at room temperature.

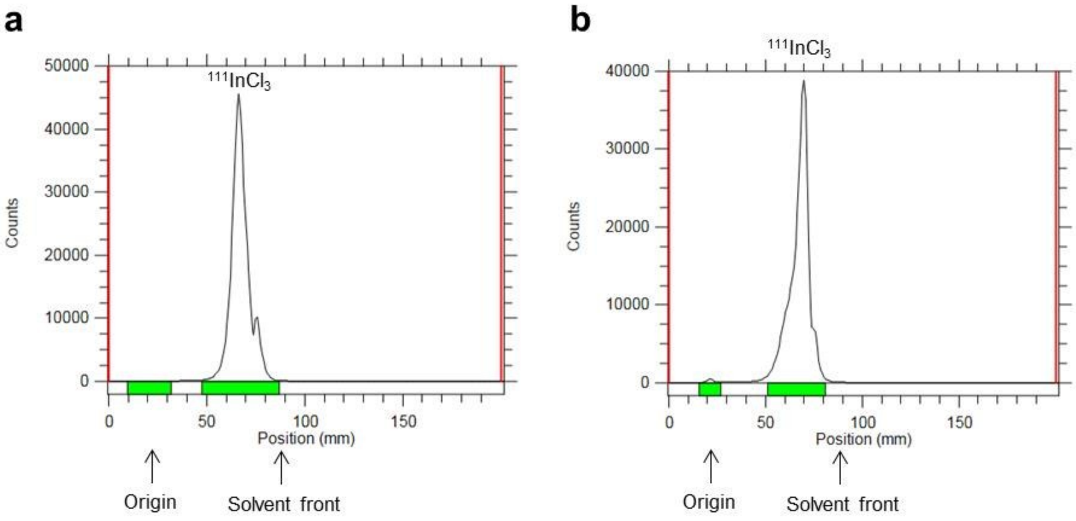

Figure S3 a) iTLC of $^{111}InCl_3$ in citrate buffer using EDTA (0.5 M, pH = 7.6) as the mobile phase. b) iTLC of PLGA-**Ru1** nanoparticles after addition of ^{111}In, indicating no nanoparticle-bound ^{111}In for the non-targeted nanoparticles.

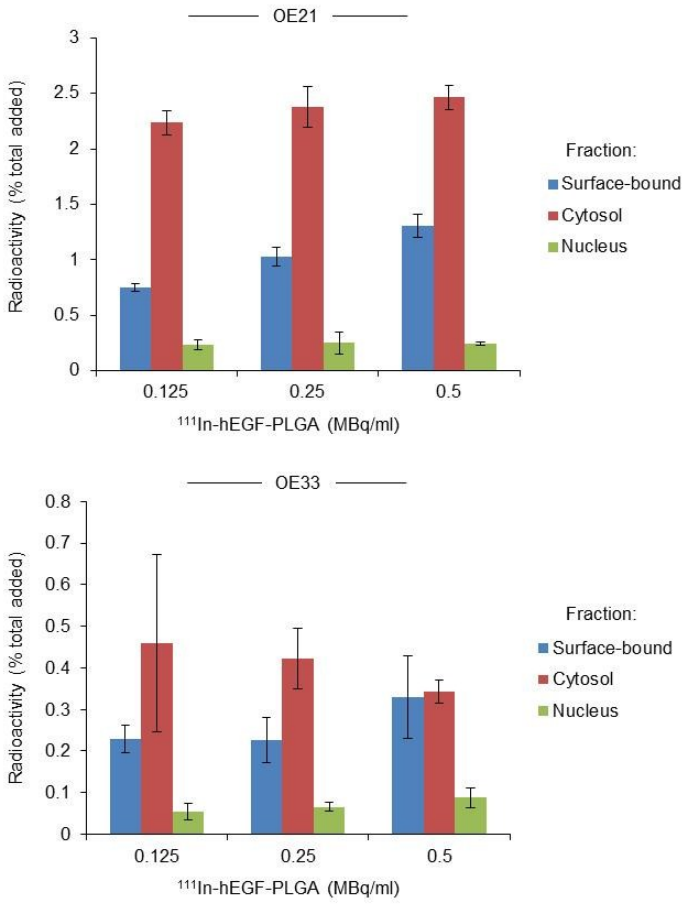

Figure S4 Data from Figure 3a expressed as % of total radioactivity added to cells and including the surface-bound component.

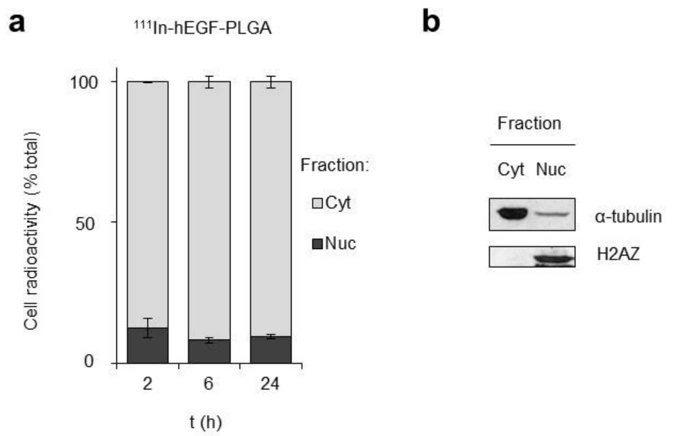

Figure S5 a) Sub-cellular radioactivity content of OE21 cells treated with ^{111}In-hEGF-PLGA (0.5 MBq/mL) for 2, 6 or 24 h. Isolated cytosol (Cyt) and nuclear (Nuc) fractions were obtained and internalisation determined by measuring radioactivity; radioactivity was normalised to fraction protein content. Data expressed as a percentage of total internalised radioactivity. Experiment performed in triplicate +/- S.D. b) Successful fractionation of cytosol and nuclei was confirmed by immunoblotting using α-tubulin and H2AZ antibodies for cytosol- and nuclei-enriched fractions respectively.

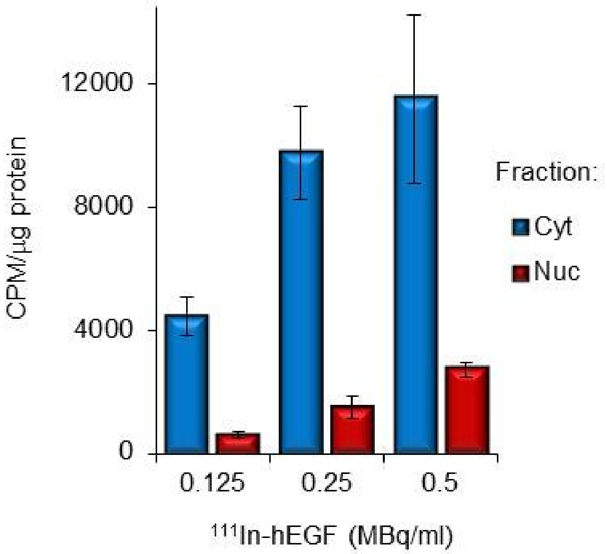

Figure S6 Sub-cellular radioactivity content of OE21 cells treated with ^{111}In-hEGF (0.125 - 0.5 MBq/mL, 2 h). Isolated cytosol (Cyt) and nuclear (Nuc) fractions were obtained. The amount of accumulated radioactivity was measured by gamma-counting and normalised to protein content of each fraction (experiment performed in triplicate +/- S.D.).

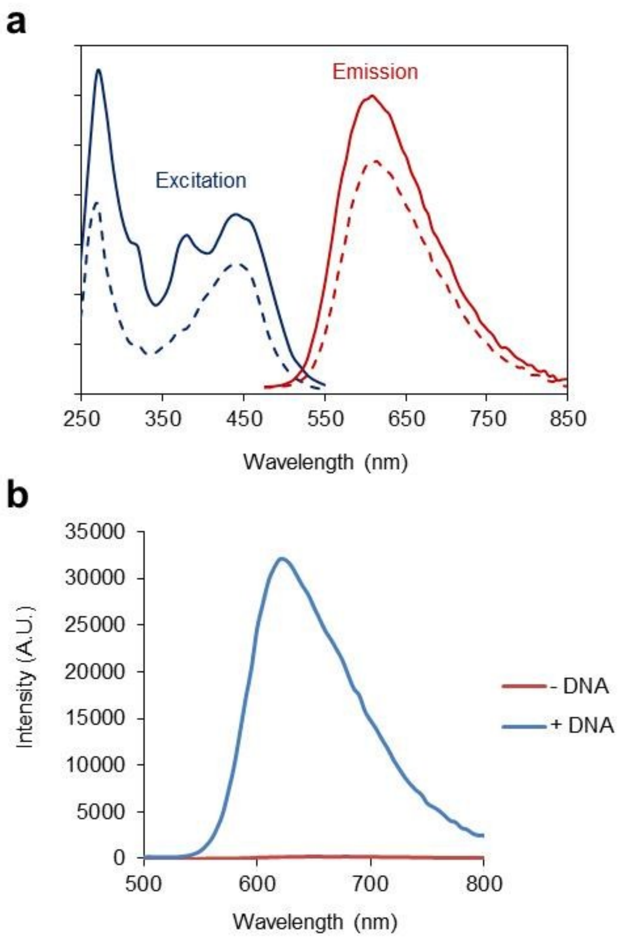

Figure S7 a) Excitation (λ_{em} = 630 nm, blue) and emission (λ_{ex} = 450 nm, red) spectra of hEGF-PLGA-**Ru1** (solid lines) in PBS. Spectra of equivalent concentration of **Ru1** included for comparison (dashed lines). b) Emission spectra (λ_{ex}=458 nm) of **Ru1** (20 μM) and **Ru1** + calf thymus DNA (20 ng/mL). Identical settings were used to collect each spectrum.

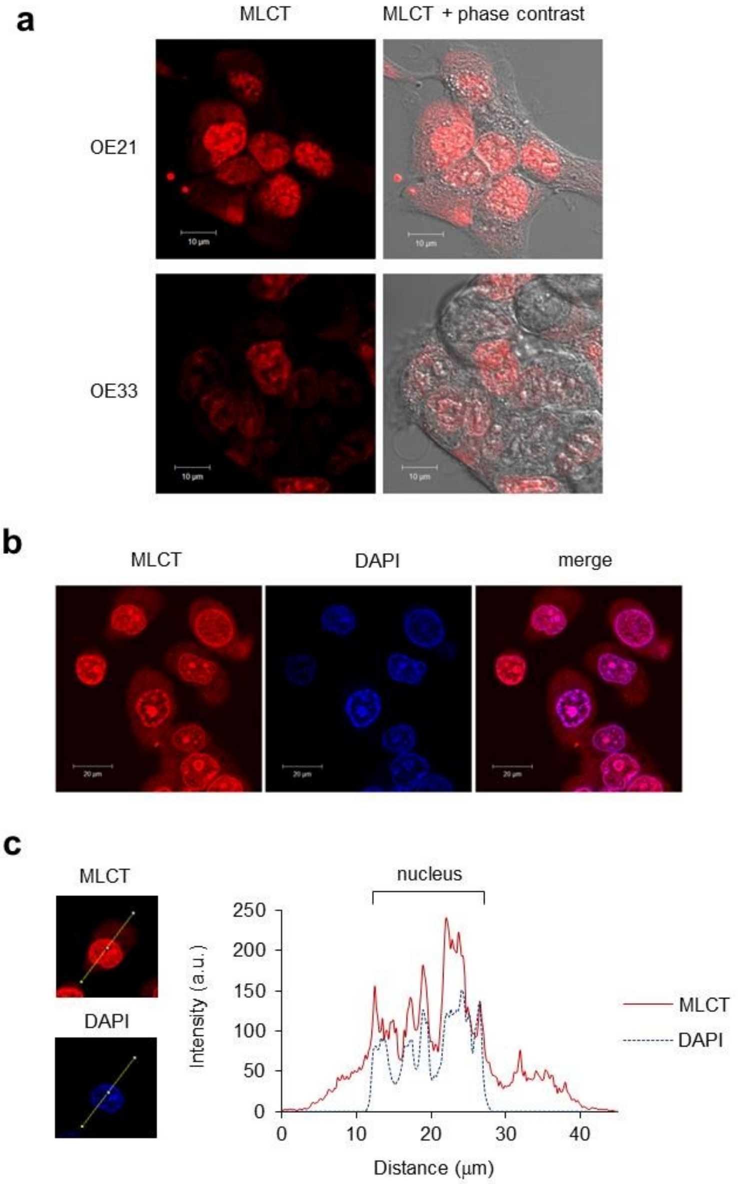

Figure S8 a) Confocal microscopy (CLSM) of OE21 or OE33 cells treated with hEGF-PLGA-**Ru1** (1 mg/mL, 24 h) showing intracellular metal to ligand charge-transfer (MLCT) emission of **Ru1**. b) CLSM of OE21 cells treated with hEGF-PLGA-**Ru1** (1 mg/mL, 24 h) co-stained with DNA dye DAPI. Cells were fixed with formaldehyde after hEGF-PLGA-**Ru1** incubation and before DAPI staining. c) Emission profiles of MLCT (red) and DAPI (blue) signals.

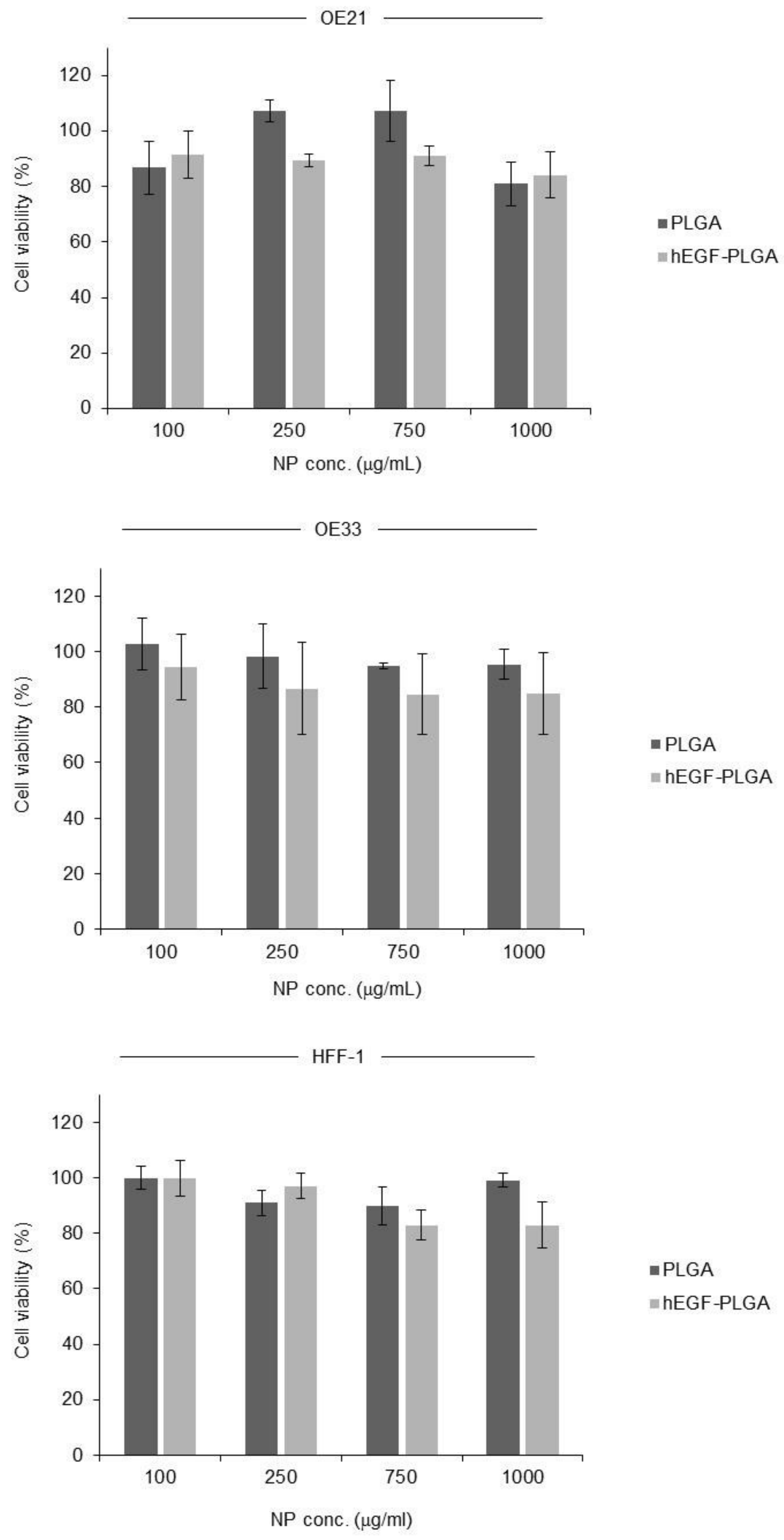

Figure S9 Impact of hEGF-PLGA or PLGA nanoparticles (NPs) on cell viability of OE21, OE33 or HFF-1 cells, as determined by MTT assay (24 h incubation). Mean of triplicates +/- S.D.

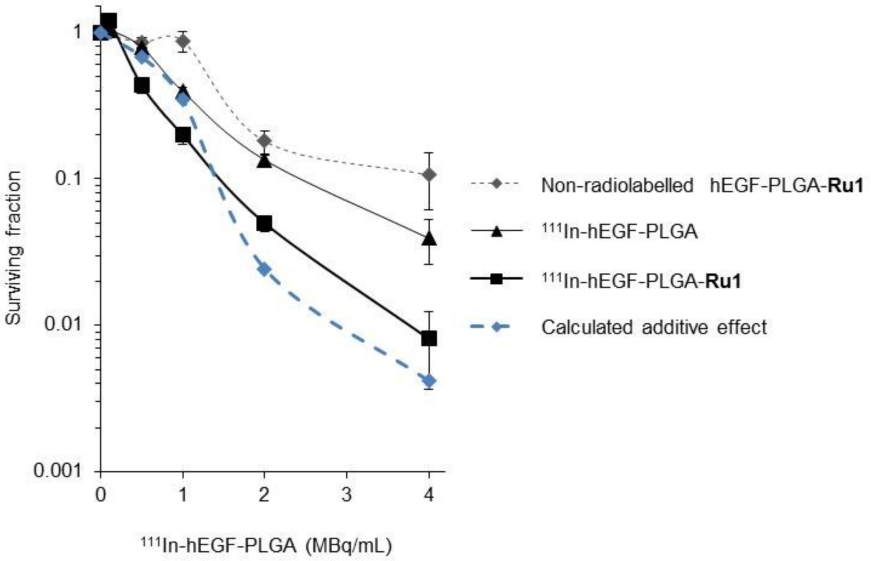

Figure S10 Data from Table S5 with a calculated additive effect of ^{111}In radiolabelling and **Ru1**-loading. Calculated added effect = S.F. of ^{111}In-hEGF-PLGA x S.F. of non-radiolabelled hEGF-PLGA-**Ru1**.

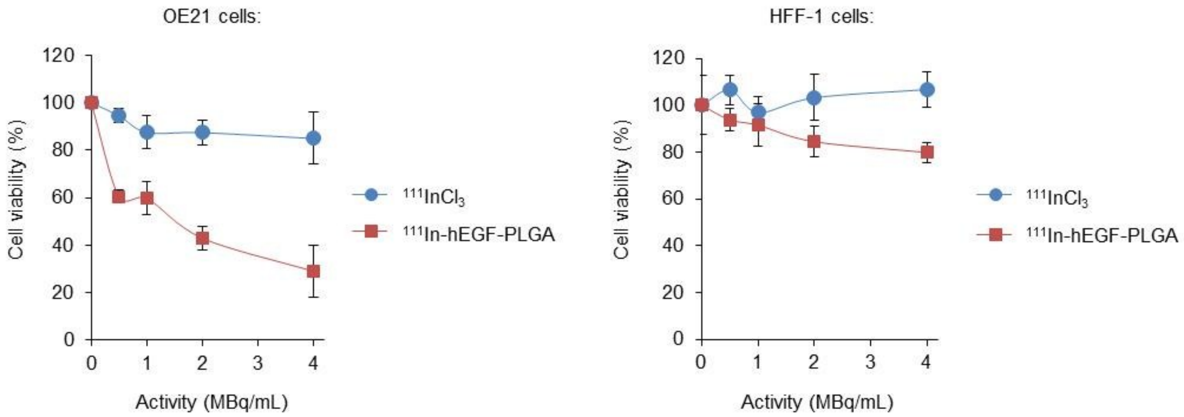

Figure S11 Impact of free $^{111}InCl_3$ or radiolabelled ^{111}In-hEGF-PLGA nanoparticles on cell viability of OE21 or HFF-1 cells. Cells were treated for 24 h with radiolabelled nanoparticles and cell viabilities determined by MTT assay 24 h post-incubation.

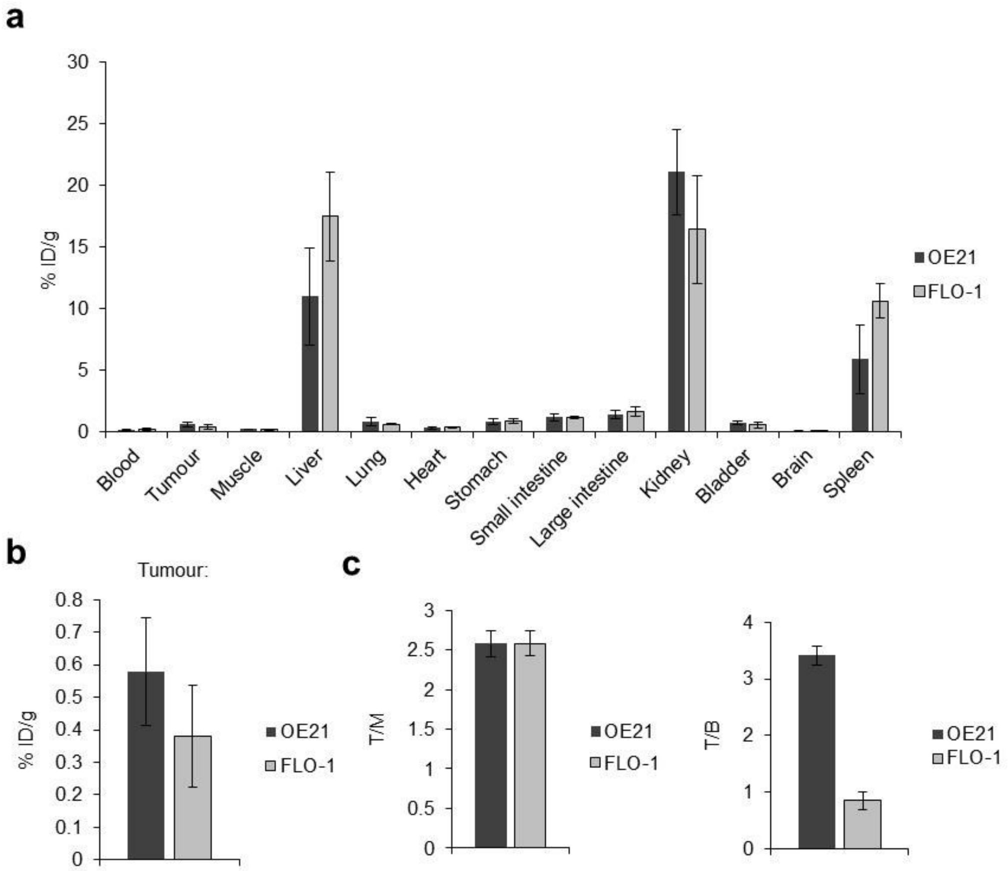

Figure S12 a) Biodistribution 24 h after i.v. administration of ^{111}In-hEGF-PLGA nanoparticles in BALB/c nude mice bearing OE21 (n=4) or FLO-1 (n=3) tumour xenografts. Results are expressed as mean +/- S.E.M. percent injected dose (radioactivity) per gram (%ID/g) values (decay corrected). b) Tumour %ID/g. c) Tumour/muscle and tumour/blood ratios. T = tumour, M = muscle, B = blood.

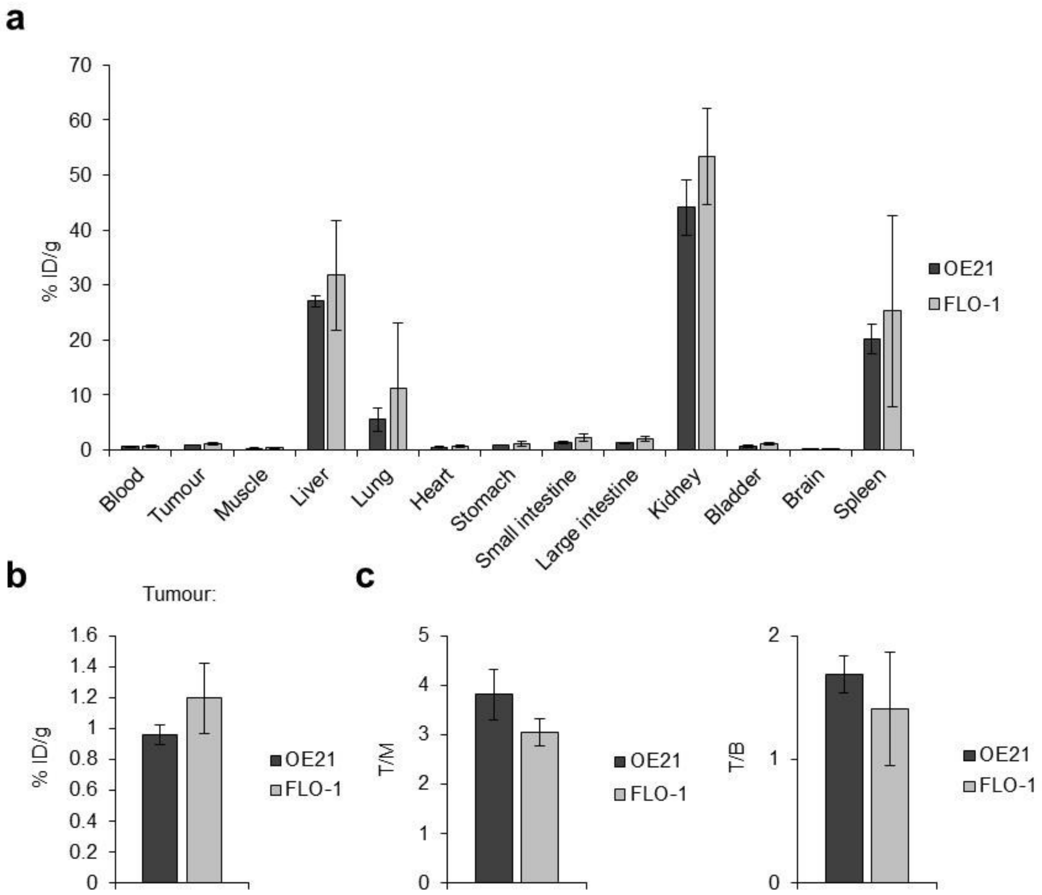

Figure S13 Biodistribution 24 h after i.v. administration of ^{111}In-hEGF-PLGA-**Ru1** nanoparticles in BALB/c nude mice bearing OE21 (n=4) or FLO-1 (n=4) tumour xenografts. Results are expressed as mean +/- S.E.M. percent injected dose (radioactivity) per gram (%ID/g) values (decay corrected). b) Tumour %ID/g. c) Tumour/muscle and tumour/blood ratios. T = tumour, M = muscle, B = blood.

References

1. M. R. Gill, P. J. Jarman, S. Halder, M. G. Walker, H. K. Saeed, J. Thomas, C. Smythe, K. Ramadan and K. Vallis, Chem. Sci., 2018, **9**, 841-849
